# Generation of proliferative hESC-derived grape-clustered hepatocyte organoids with multipolar architecture as regenerative counterpart via synergy of YAP and IGF2 pathways

**DOI:** 10.1038/s41419-026-08635-y

**Published:** 2026-03-26

**Authors:** Haibin Wu, Shoupei Liu, Sen Chen, Changlu Qin, Wenjiao Yan, Xiangting Cao, Yongjian Zhou, Yuyou Duan

**Affiliations:** 1 Department of Gastroenterology and Hepatology, Guangzhou Digestive Disease Center, the Second Affiliated Hospital, School of Medicine, South China University of Technology, Guangzhou, China; 2 Laboratory of Stem Cells and Translational Medicine, Center for Medical Research on Innovation and Translation, Institute of Clinical Medicine, the Second Affiliated Hospital, School of Medicine, South China University of Technology, Guangzhou, China; 3 Laboratory of Stem Cells and Translational Medicine, Institute for Life Science, School of Medicine, South China University of Technology, Guangzhou, China; 4 National Engineering Research Center for Tissue Restoration and Reconstruction, South China University of Technology, Guangzhou, China; 5 The Innovation Centre of Ministry of Education for Development and Diseases, the Second Affiliated Hospital, School of Medicine, South China University of Technology, Guangzhou, China

**Keywords:** Embryonic stem cells, Differentiation, Translational research

## Abstract

Primary human hepatocyte (PHH)-derived organoids form grape-like clusters with proliferative capacity, hepatocyte functionality, and multipolar polarity, serving as valuable models for liver biology and therapeutics. However, deriving comparable organoids from human embryonic stem cells (hESCs) remains difficult. Here, we established a defined system to differentiate hESC-derived hepatoblast organoids into hepatocyte organoids (heporgs) with two morphologies: spheroid-like (S-heporgs) and grape-like (G-heporgs). S-heporgs predominated but displayed senescence and apoptosis, generating an inflammatory niche that facilitated G-heporg emergence. G-heporgs exhibited mature hepatocyte markers, binucleation, proliferative activity, and multipolar structures with branched bile canaliculi, closely resembling PHH-derived organoids. Transcriptomic and functional analyses identified IGF2-driven PI3K-AKT activation as essential for G-heporg formation, while YAP signaling supported their long-term expansion. IGF2 supplementation combined with YAP agonist treatment enabled stable G-heporg propagation for over 60 days. These expandable G-heporgs demonstrated regenerative competence and faithfully recapitulated hepatocyte polarity and functional bile canalicular networks, as evidenced by ATP7B copper-dependent translocation and drug-induced cholestasis assays. Our findings establish hESC-derived G-heporgs as expandable, functional counterparts to PHH-derived organoids, providing a robust platform for studying hepatocyte polarity, metabolite trafficking, and liver disease modeling.

## Introduction

The liver is an essential organ responsible for diverse functions, including metabolism, digestion, protein synthesis, and detoxification [1]. Hepatocytes, the predominant parenchymal cell type, carry out these critical functions [2]. In vitro culture of mature hepatocytes holds great promise for studies on hepatocyte development, drug screening, and clinical applications [3, 4]. However, maintaining functional hepatocytes in long-term culture remains challenging, as they undergo rapid dedifferentiation characterized by diminished metabolic and propagation capacity [5].

With advances in organoid technology, human liver tissue-derived organoids are increasingly utilized to replace traditional culture systems [6]. Specifically, cholangiocyte organoids derived from LGR5^+^ biliary epithelial progenitor cells exhibit cystic morphology, long-term expansion potential, and bipotent differentiation ability [7]. On the other hand, grape-like hepatocyte organoids (heporgs), generated from human or mouse primary mature hepatocytes [8–10], exhibit moderate proliferative capacity. Although their expansion potential is lower than that of cholangiocyte organoids, heporgs retain strong hepatic functionality [9, 10]. Crucially, heporgs display multipolar architectures with one or more apical poles contributing to bile canaliculi (BC), more faithfully reproducing the polarity of hepatocytes in vivo [11]. This structural fidelity enables improved modeling of directional protein and metabolite trafficking. Conversely, cholangiocyte organoids form typical columnar polarity resembling that of most epithelial cells [7].

The limited supply and variability of human primary liver tissues from both healthy and diseased donors restrict the large-scale production of hepatocyte organoids for research and clinical use [12]. Human pluripotent stem cells (hPSCs), with their self-renewal and multilineage differentiation potential, represent a promising alternative [13–15]. However, current hPSC-derived hepatoblast organoids resemble cholangiocyte organoids [15], and generating proliferative grape-like heporgs with multipolar architecture from hPSCs remains a major challenge.

In this study, we established a defined culture system that enabled the generation of mature grape-like heporgs (G-heporgs) from human embryonic stem cells (hESCs). Within this culture system, spherical hepatocyte organoids (S-heporgs) predominated, generating a liver injury-associated inflammatory microenvironment that promoted the emergence of G-heporgs, recapitulating key features of the hepatocyte regeneration process. Similar to primary hepatocyte-derived heporgs, G-heporgs exhibited high albumin (ALB) expression and expansion potential, whereas S-heporgs lacked these properties. Comparative analyses identified Insulin-like Growth Factor 2 (IGF2) as a driver of G-heporg formation. Moreover, targeted modulation of the Hippo–YAP pathway overcame long-term expansion barriers in G-heporgs. Notably, expandable G-heporgs as a regeneration counterpart developed multipolar structures with functional BC networks, closely recapitulating primary hepatocyte polarity. Finally, we demonstrated the application of G-heporgs in modeling copper ion metabolism, validating their utility for investigating polarized metabolite trafficking in hepatocytes.

## Results

### Differentiation of G-heporgs from hESC-derived HB-orgs

**Fig. 1:**
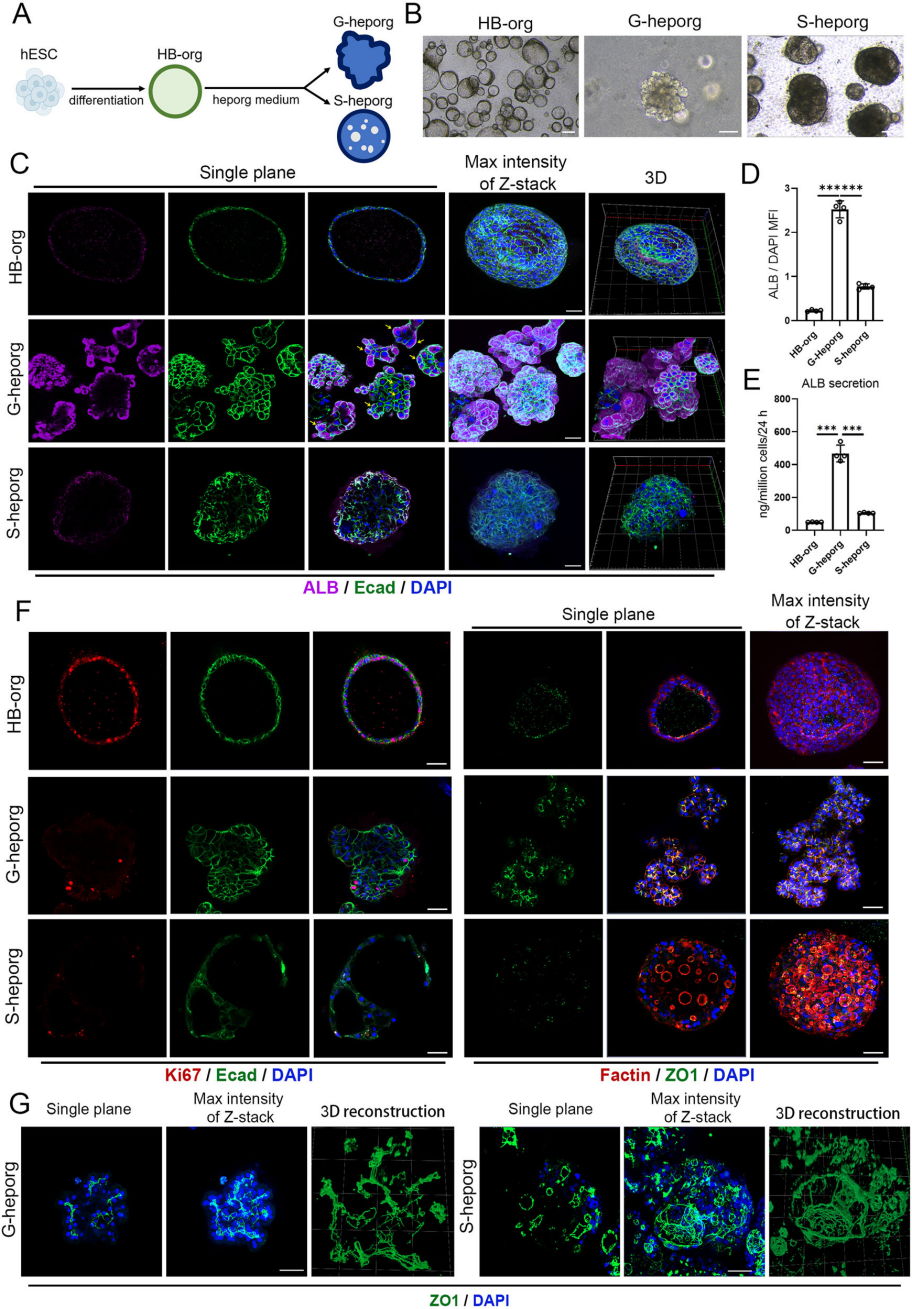
Generation and characterization of G-heporgs from hESCs. **A** Schematic illustration of the generation of G-heporgs and S-heporgs from hESC-derived HB-orgs. **B** Representative morphologies of HB-orgs, G-heporgs and S-heporgs. Scale bar = 100 μm. **C** Immunostaining of ALB and Ecad in indicated groups. Nuclei were stained with DAPI. Yellow arrows indicated binucleated hepatocytes. Scale bar = 50 μm. **D** The quantification of ALB expression intensity. *n* = 4 biologically independent experiments. **E** The analysis of the secretion of ALB in indicated groups. *n* = 4 biologically independent experiments. **F** Immunostaining of Ki67, Ecad, Factin, and ZO1 in indicated groups. Nuclei were stained with DAPI. Scale bar = 50 μm. **G** Representative immunostaining and 3D reconstruction images for ZO1 in indicated groups. Nuclei were stained with DAPI. Scale bar = 50 μm. Results were presented as mean ± SD. Statistical significance was determined using one‑way ANOVA followed by Tukey post‑test. ***p < 0.001.

In our previous study, we established hepatoblast organoids (HB-orgs) derived from hESCs and demonstrated their bipotent differentiation potential, long-term expansion capacity, and hepatic progenitor characteristics [15]. To generate mature hepatocyte organoids (heporgs) from hESCs, we started with culturing HB-orgs in heporg medium (Fig. 1A). After prolonged culture, two distinct organoid morphologies unexpectedly emerged: grape-like heporgs (G-heporgs), forming clusters similar to PHH-derived organoids [8, 9], and sphere-like heporgs (S-heporgs) (Figs. 1B and S1a, b). G-heporgs expanded into large organoids up to 600 μm in diameter, whereas S-heporgs remained at 100–300 μm. Notably, some G-heporgs appeared as budding structures emerging from S-heporgs (Fig. S1c). Based on these clear morphological differences, G-heporgs and S-heporgs were readily separated.

Consistent with PHH-derived heporgs [8, 9], differentiated G-heporgs showed markedly elevated albumin (ALB) expression and secretion compared with HB-orgs and S-heporgs (Fig. 1C–E). RT-qPCR confirmed the upregulation of key hepatocyte function-related genes, including *ALB*, *SERPINA1* (*α1AT*), *CYP2C9*, *CYP3A4*, and *ABCB11* (*BSEP*) (Figure S1d). Additionally, immunofluorescence (IF) staining clearly identified numerous binucleated hepatocytes in G-heporgs, a hallmark feature of mature hepatocytes (Fig. 1C), as well as partial expression of the proliferation marker Ki67 (Fig. 1F). These organoids displayed organized cellular architecture with strong expression of epithelial marker E-cadherin (E-cad) and tight junction protein ZO1 (Fig. 1C, F). In contrast, S-heporgs displayed disorganized cell structures, low ALB expression, and an absence of Ki67 (Fig. 1C, F).

ZO1 staining at day 21 revealed distinct polarization patterns: G-heporgs developed a branched three-dimensional bile canaliculi (BC) network closely resembling in vivo liver architecture [11, 16], confirming a well-defined multipolar structure (Fig. 1G). In contrast, although S-heporgs also expressed ZO1, they failed to form a functional canalicular network (Fig. 1G). Collectively, these findings demonstrate the successful generation of G-heporgs from hESCs, exhibiting features consistent with PHH-derived heporgs, including robust expression of mature hepatocyte genes, grape-like morphology, proliferative potential, and multipolar organization.

### Identification of divergent cellular states associated with G-heporg emergence

**Fig. 2:**
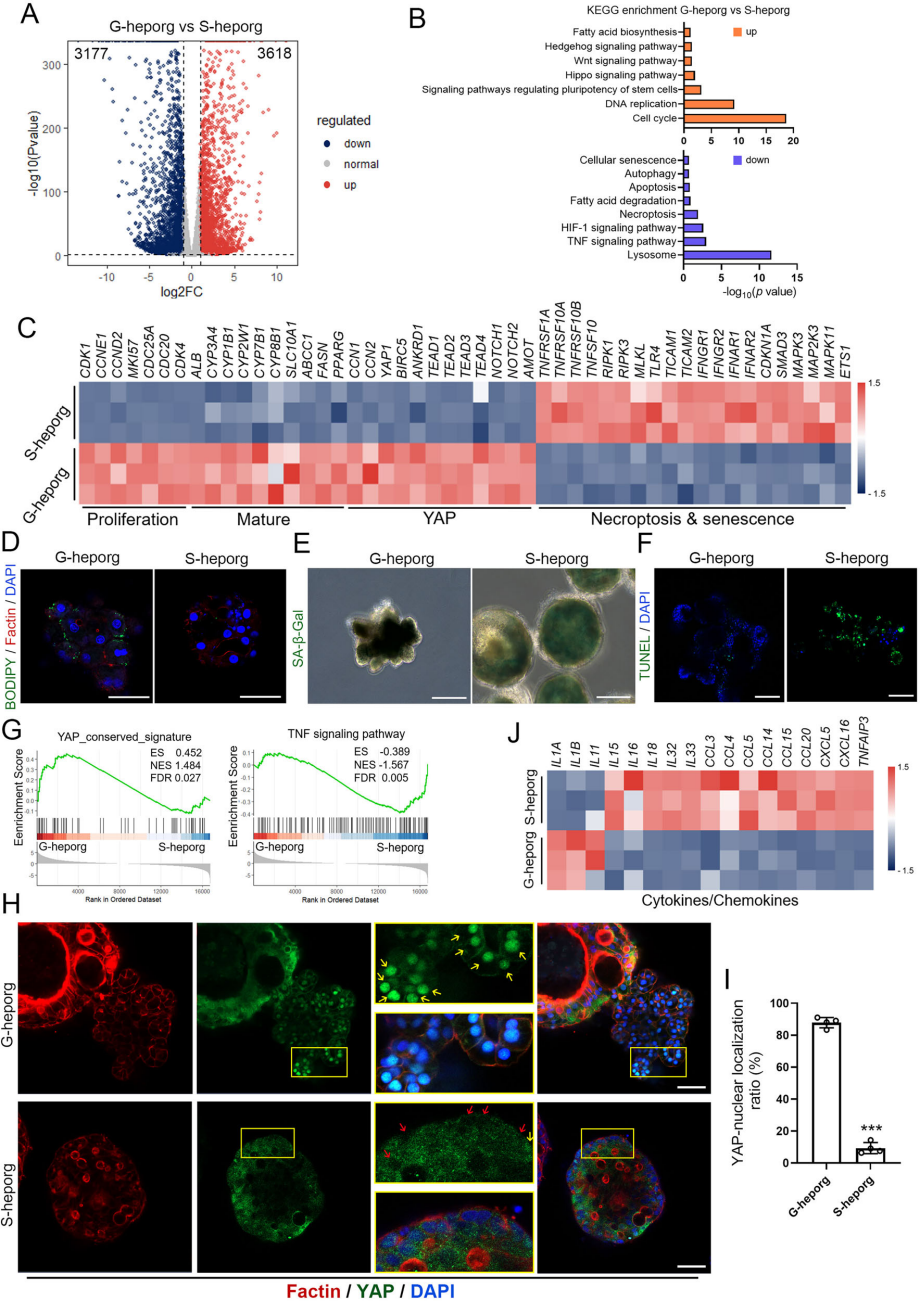
Comparison between G-heporgs and S-heporgs. **A** Volcano plot analysis of G-heporgs and S-heporgs. **B** Analysis of KEGG pathway of G-heporgs versus S-heporgs. **C** Heatmap of G-heporgs and S-heporgs for genes related to proliferation, mature, YAP, and necroptosis and senescence. **D** Immunostaining of Factin and lipid droplet (BODIPY staining) in indicated groups. Nuclei were stained with DAPI. Scale bar = 50 μm. **E** Analysis of cell senescence by SA-β-Gal staining. Scale bar = 100 μm. **F** Analysis of cell apoptosis by TUNEL staining. Nuclei were stained with DAPI. Scale bar = 100 μm. **G** GSEA analysis. **H** Immunostaining of Factin and YAP in indicated groups. Nuclei were stained with DAPI. Scale bar = 50 μm. Yellow arrows indicate YAP with nuclear localization, red arrows indicated YAP without nuclear localization. **I** Quantification of YAP nuclear localization. *n* = 4 biologically independent experiments. **J** Heatmap of G-heporgs and S-heporgs for genes related to cytokines and chemokines. Results were presented as mean ± SD. Statistical significance was determined using unpaired two-tailed Student’s *t* test. ***p < 0.001.

We found it intriguing that two distinct organoid types, G-heporgs and S-heporgs, emerged from the same batch of HB-orgs cultured under identical conditions. To elucidate the reason of G-heporg generation, we performed bulk RNA sequencing (RNA-seq) analysis on both organoid types. Approximately 6,795 differentially expressed genes (DEGs) were identified between G-heporgs and S-heporgs (Fig. 2A). Kyoto Encyclopedia of Genes and Genomes (KEGG) pathway analysis revealed significant enrichment related to cell cycle, DNA replication and other pathways which were benefit to hepatocyte proliferation in G-heporgs. In contrast, S-heporgs were enriched in pathways associated with HIF-1 signaling, necroptosis, apoptosis and cellular senescence (Fig. 2B). Heatmap analysis further confirmed that S-heporgs highly expressed genes associated with hepatocyte necrosis and senescence, while G-heporgs displayed elevated expression of genes involved in cell proliferation and hepatocyte maturation (Fig. 2C). Proliferating hepatocytes preferentially engage in lipid synthesis rather than catabolic metabolism [17, 18]. Consistently with this, G-heporgs expressed high levels of fatty acid synthesis–related genes (Fig. S2a, b) and showed substantial intracellular lipid droplet accumulation determined, as revealed by BODIPY staining, compared with S-heporgs (Fig. 2D). Furthermore, senescence-associated β-galactosidase (SA-β-Gal) staining and TUNEL assays confirmed extensive cellular senescence and apoptosis in S-heporgs, which were minimal or absent in G-heporgs (Fig. 2E, F). Collectively, these transcriptomic, metabolic, and functional analyses demonstrated that G-heporgs resided in a highly proliferative and metabolically active state, whereas S-heporgs were characterized by pronounced cellular senescence and necrosis.

Notably, Heatmap and Gene Set Enrichment Analysis (GSEA) analyses demonstrated strong upregulation of YAP signaling pathway in G-heporgs (Fig. 2C, G), a pathway essential for organ development and liver regeneration [19, 20]. Although normally inactive in mature hepatocytes [21], Yap signaling is reactivated during liver regeneration after severe injury, where inflammatory cues promote hepatocyte regeneration and proliferation [22, 23]. Nuclear localization of YAP serves as a hallmark of pathway activation [20]. To validate this activation, we performed IF assay and identified a hybrid organoid that G-heporg budded from S-heporg. Strikingly, YAP was predominantly localized in the nuclei of G-heporgs, but rarely in adjacent S-heporgs (Fig. 2H, I). Consistently, Yap target genes were highly expressed in G-heporgs, confirming the elevated Yap activity (Fig. 2C). Multiple inflammatory factors have been reported to promote the expansion of PHH-derived heporgs [10, 17]. We therefore hypothesized that long-term cultured S-heporgs generate a hepatocyte injury-associated microenvironment, which drives the emergence of G-heporgs as a regenerative product. Consistent with this hypothesis, S-heporgs exhibited pronounced cellular senescence and apoptosis, together with elevated expression of pro-inflammatory mediators, including TNF and cytokine-related genes (Fig. 2G, J), resembling transcriptional profiles observed in acute liver failure (Fig. S2c). Moreover, analysis of highly expressed inflammatory factors confirmed a gradual increase in the secretion of C-C motif chemokine ligand 20 (CCL20) and Interleukin 32 (IL32) in the culture system, indicating the presence of a sustained inflammatory milieu during long-term culture (Fig. S2d). Together, these results demonstrated that G-heporgs existed in a proliferative state resembling hepatocyte regeneration, whereas S-heporgs displayed senescence and necrosis, and the inflammatory microenvironment generated by S-heporgs might have contributed to the induction of G-heporgs.

### IGF2 promoted the generation and expansion of G-heporgs

**Fig. 3:**
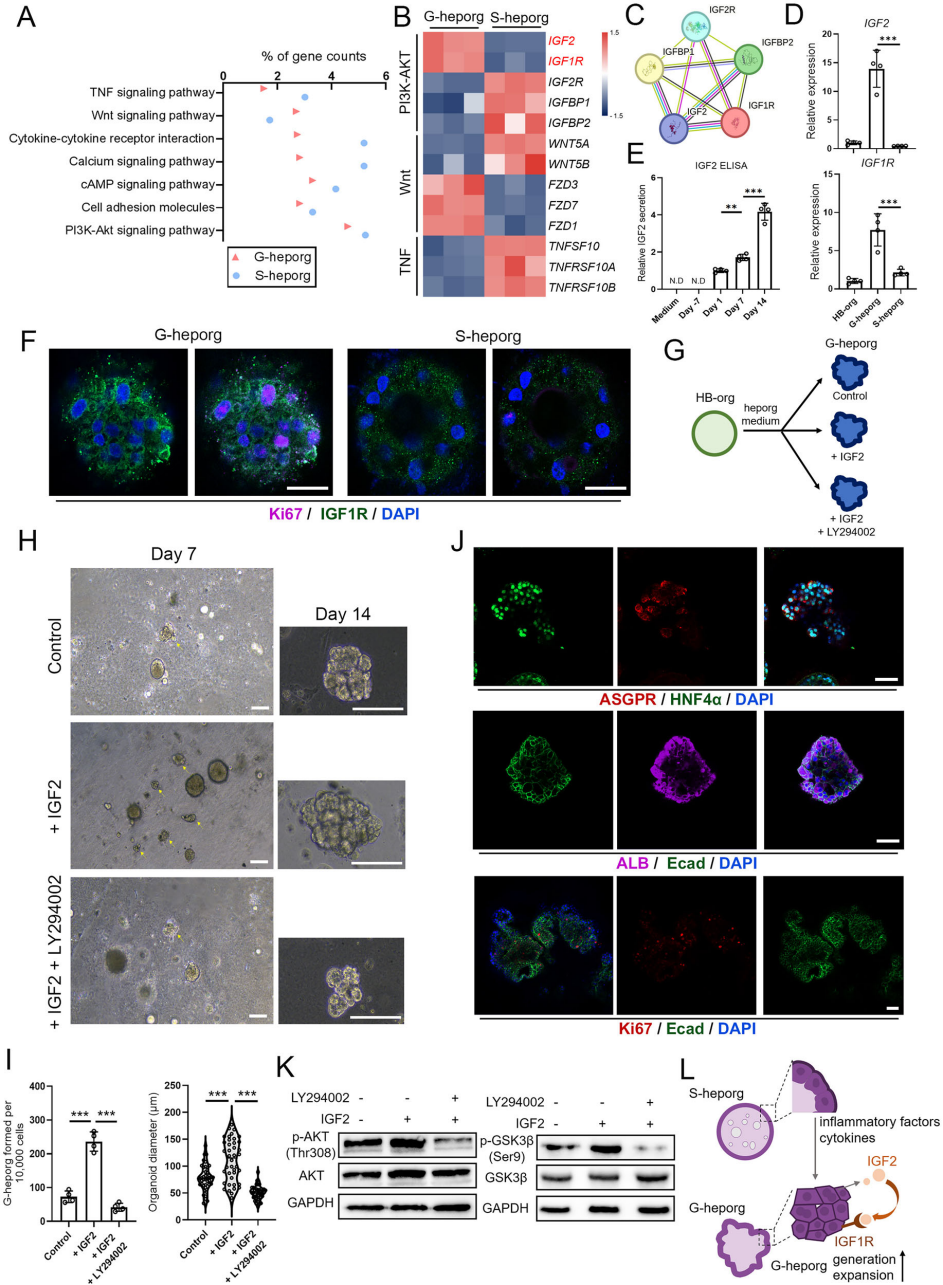
IGF2 promoted the generation and expansion of G-heporgs. **A** KEGG pathway analysis of G-heporgs and S-heporgs. **B** Heatmap showing ligands and receptors in G-heporgs and S-heporgs. **C** Matched signaling of IGF2 and IGF1R using STRING database. **D** Expressions of IGF2 and IGF1R were analyzed by RT-qPCR in different organoids. *n* = 4 biologically independent experiments. **E** IGF2 secretion from culture medium at different days (day 1 was the first day that observed the generation of G-heporgs). *n* = 4 biologically independent experiments. **F** Immunostaining for IGF1R and Ki67. Nuclei were stained with DAPI. Scale bar = 25 μm. **G** Experimental strategy to evaluate the effect of exogenous IGF2 and LY294002 (PI3K-AKT inhibitor) on G-heporgs. **H** Representative morphologies of G-heporgs after the treatment with or without IGF2 and LY294002 at different days. Scale bar = 100 μm. **I** Quantification of the formation efficiency (*n* = 4 biologically independent experiments) and diameter (*n* = 40 organoids) of G-heporgs at day 14 after different treatments. **J** Immunostaining of ASGPR, HNF4α, ALB, Ecad and Ki67 for G-heporgs treated with IGF2. Nuclei were stained with DAPI. Scale bar = 50 μm. **K** Western blot assessment for the expression of p-AKT(Thr308), AKT, p-GSK3β(Ser9) and GSK3β in G-heporgs after different treatments. **L** Schematic illustration of cell-cell interactions between G-heporgs and S-heporgs. Results were presented as mean ± SD. Statistical significance was determined using one‑way ANOVA followed by Tukey post‑test. *p < 0.05, **p < 0.01, ***p < 0.001.

To identify key factors responsible for inducing G-heporgs formation in our culture system, we focused on receptor-ligand interactions using transcriptomic sequencing data by comparing G-heporgs and S-heporgs (Fig. 3A). The results revealed significant upregulation of insulin-like growth factor 2 (*IGF2*) and its receptor insulin-like growth factor 1 receptor (*IGF1R*) in G-heporgs (Fig. 3B). Binding of IGF2 to IGF1R activates PI3K-AKT signaling pathway, which is critical for cell proliferation [24]. In hepatocytes, IGF2 functions as a critical regulator in liver repopulation and is induced by inflammatory signals [25, 26]. We therefore hypothesized that inflammatory cytokines secreted by S-heporgs upregulated IGF2 expression, thereby driving the generation of neighboring G-heporgs. The interaction between IGF2 and IGF1R was validated using CellTalkDB [27] and STRING database [28] (Fig. 3C). Although IGF2 can bind to IGF2R as well as to Insulin-like Growth Factor Binding Proteins 1 and 2 (IGFBP1 and IGFBP2), which antagonize its activity [29], these inhibitory genes were expressed at low levels in G-heporgs (Fig. 3B). RT-qPCR validated elevated expression of *IGF2* and *IGF1R* in G-heporgs (Fig. 3D), and both IF analysis and Enzyme-Linked Immunosorbent Assay (ELISA) verified robust protein expression (Fig. 3E, F).

To investigate the role of IGF2 in driving G-heporg formation and expansion, we supplemented the medium with IGF2 and concurrently treated organoids with the LY294002 (PI3K-AKT inhibitor), as IGF2 is known to activate PI3K-AKT signaling (Fig. 3G). IGF2 treatment markedly increased both the formation rate and growth of G-heporgs, yielding substantially larger organoids by day 14 (Fig. 3H, I). In addition, IGF2-treated G-heporgs maintained strong expression of ALB, HNF4α, and E-cad, with partial expression of ASGPR and Ki-67 (Fig. 3J). RT-qPCR further confirmed increased *Ki67* expression without changes in hepatocyte-specific gene expression (Fig. S3a). Co-treatment with IGF2 and LY294002 inhibited PI3K-AKT signaling, as shown by decreased p-AKT and p-GSK3β levels (Figs. 3K and S3b). This led to a significant reduction in both G-heporg formation rates and organoid sizes (Fig. 3H, I). In addition, to further examine the role of IGF2 signaling in the formation and expansion of G-heporgs, an IGF1R inhibitor (Linsitinib) was added to the culture medium. We found that Linsitinib not only significantly reduced organoid diameters but also completely blocked the formation of G-heporgs (Fig. S3c–e). Collectively, these results demonstrate that the binding of IGF2 to IGF1R is essential for G-heporg formation and growth, acting through PI3K-AKT signaling pathway.

To determine whether IGF2 expression in G-heporgs was directly associated with S-heporgs, G-heporgs were isolated from S-heporgs and cultured independently. We found that *IGF2* expression in G-heporgs gradually declined upon serial passaging (Fig. S4a). However, the supplementation of independently cultured G-heporgs with 25% S-heporg-conditioned medium (S-CM) markedly promoted G-heporg proliferation (Fig. S4b, c) and significantly increased *IGF2* expression (Fig. S4d), indicating that *IGF2* expression in G-heporgs is closely linked to signals derived from S-heporgs. In contrast, during the differentiation of G-heporgs from HB-orgs, the supplementation with S-CM failed to enhance G-heporg formation efficiency and instead completely blocked G-heporg generation (Fig. S4e, f). On the one hand, this might be due to the inability of S-CM to induce *IGF2* expression in HB-orgs (Fig. S4d). On the other hand, direct treatment of HB-orgs with S-CM may not recapitulate the strict temporal requirement for inflammatory niche signals during G-heporg differentiation.

Collectively, these results demonstrated that the inflammatory environment generated by S-heporgs contributes to enhanced IGF2 expression in G-heporgs. IGF2 subsequently promotes the generation and expansion of G-heporgs through IGF1R-mediated activation of PI3K–AKT pathway (Fig. 3L).

### Activation of Yap signaling promoted long-term expansion of G-heporgs

**Fig. 4:**
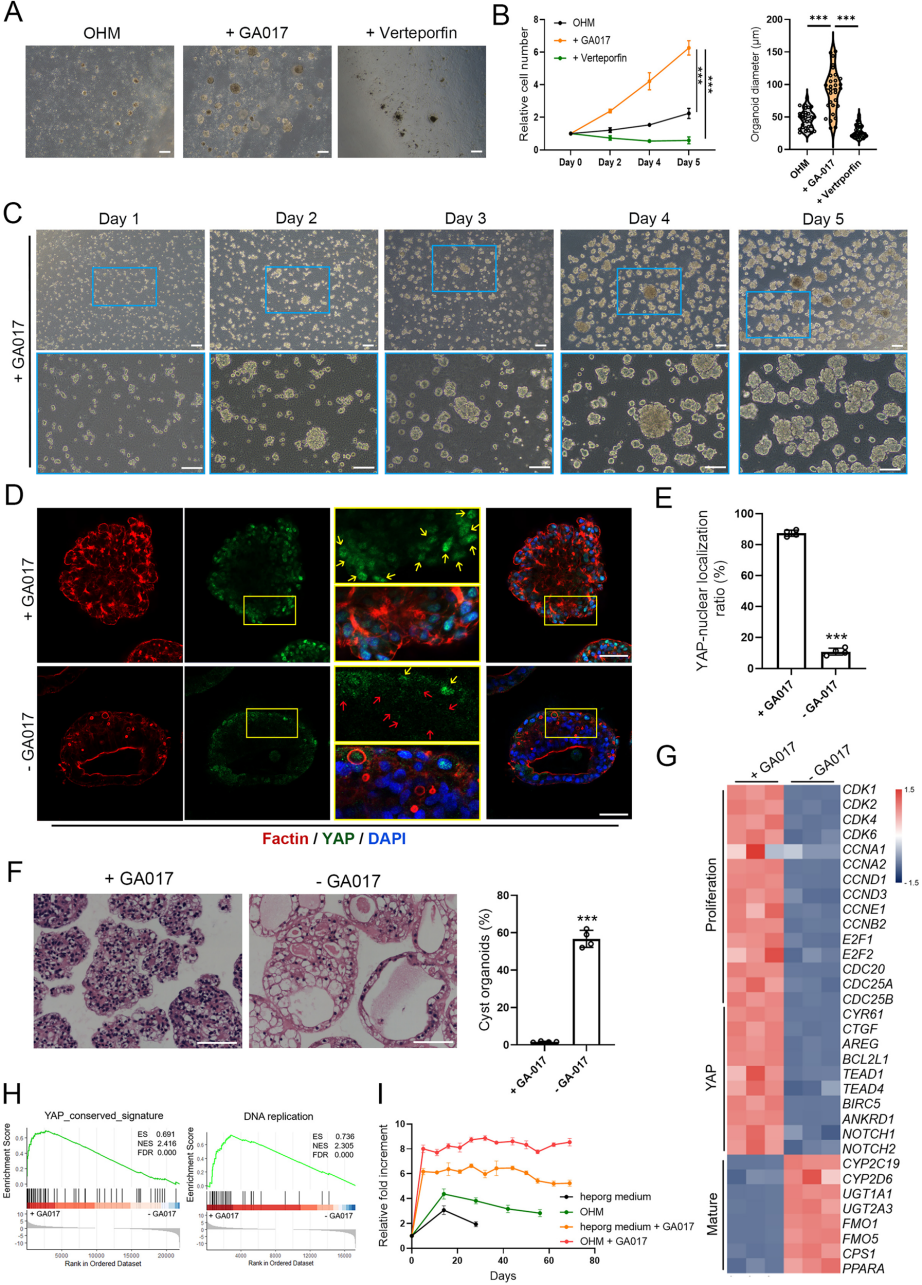
Long-term culture of G-heporgs through the regulation of YAP signaling. **A** Representative images of G-heporgs after the treatment with YAP agonist (GA017) or inhibitor (Verteporfin) in optimized heporg medium (OHM). **B** Relative cell number (*n* = 4 biologically independent experiments) and organoid diameter (*n* = 30 organoids) of G-heporgs at day 5 after the treatment. **C** Representative morphologies of G-heporgs after the treatment with GA017 at different days. Scale bar = 100 μm. **D** Immunostaining of Factin and YAP. Nuclei were stained with DAPI. Scale bar = 50 μm. Yellow arrows indicate YAP with nuclear localization, red arrows indicate YAP without nuclear localization. **E** The quantification of YAP nuclear localization. *n* = 4 biologically independent experiments. **F** H&E staining for G-heporgs treated with or without GA017, and the quantification of the percentage of cysts in organoids. Scale bar = 100 μm. *n* = 4 biologically independent experiments. **G** Heatmap of G-heporgs treated with or without GA017 for genes related to proliferation, mature and YAP. **H** GSEA analysis. **I** The relative fold increment of G-heporgs in the EM with GA017 or IGF2. Each dot represents a passage event. *n* = 4 biologically independent experiments. Results were presented as mean ± SD. Statistical significance was determined using two‑way repeated measures ANOVA followed by Tukey’s multiple comparisons test, one‑way ANOVA followed by Tukey post‑test and unpaired two-tailed Student’s t-test. ***p < 0.001.

Unlike cholangiocyte- or hepatoblast-derived organoids, PHH-derived heporgs exhibit limited long-term expansion potential in vitro [7, 15, 17]. Our differentiated G-heporgs also faced this challenge. Although IGF2 enhanced initial generation and expansion of G-heporgs, it failed to support sustained long-term propagation (Fig. S5a). Other inflammatory factors and cytokines were likewise insufficient to maintain prolonged G-heporg culture (Fig. S5a, b). To address this limitation, comparative analysis was performed in G-heporgs from passage 1 and passage 4, and we observed a marked decline in the proportion of cells showing nuclear YAP localization at passage 4 (Fig. S5c). Given the essential role of YAP signaling in hepatocyte proliferation during liver regeneration, and our prior observation that YAP signaling was markedly more active in G-heporgs than in S-heporgs (Fig. 2C, G, H), we investigated the effect of YAP signaling modulation on the expansion of G-heporgs. Strikingly, treatment with the agonist of YAP signaling (GA017) dramatically enhanced the proliferative capacity of G-heporgs, resulting in significantly larger organoids within five days while maintaining their characteristic grape-like cluster morphology (Fig. 4A–C). On the contrary, the YAP inhibitor (Verteporfin) substantially suppressed G-heporg growth (Fig. 4A–C). GA017 treatment maintained robust YAP activation (nuclear localization), whereas untreated G-heporgs displayed nuclear YAP in only a subset of cells (Fig. 4D, E). Correspondingly, GA017 treatment led to a significant upregulation of YAP target genes *CTGF* and *CYR61*, while Verteporfin treatment caused a marked decrease in their expression (Fig. S6a). Furthermore, GA017-treated G-heporgs maintained high expression of HNF4α and Ki67, partially expressed mature hepatocyte markers (ALB, α1AT) and hepatic progenitor markers (AFP, CK19), but lacked biliary progenitor marker expression (TBX3, SOX9) (Fig. S6b). Hematoxylin and eosin (H&E) staining confirmed that GA017 preserved compact organoid architecture, whereas 60% of untreated organoids became vesiculated (Fig. 4F). Moreover, RNA-seq and GSEA analyses revealed upregulation of genes involved in cell proliferation, DNA replication, and YAP target genes in GA017-treated G-heporgs (Fig. 4G, H). Combined treatment with GA017 and IGF2 enabled exponential G-heporg propagation for over 60 days and 10 passages without loss of proliferative capacity, while maintaining a normal karyotype. (Figs. 4I and S6c), establishing YAP signaling modulation as critical for long-term expandability.

To further characterize G-heporgs, transcriptome data from PHHs [8, 30], fetal hepatocytes [31–33], and human hepatocyte organoids (HHOs) [8] were integrated with our dataset. Principal-component analysis (PCA) and correlation heatmaps of the integrated dataset revealed that both early-passage and expandable G-heporgs closely resembled fetal hepatocytes (Fig. S7a, b). G-heporgs exhibited high expression of genes associated with proliferation (*IGF2*, *CCND2*), hepatoblast (*CK19*, *EPCAM*, *AFP*), and a subset of hepatocyte maturation markers (*TTR*, *APOA1*, *GSTP1*) (Fig. S7c). In contrast, PHH-derived HHOs showed high expression of hepatocyte maturation genes, consistent with their parental PHHs (Fig. S7c), indicating the advantage of the PHH-derived source for generating functionally mature organoids.

Compared with PHH-derived heporg culture systems [8, 17], we omitted forskolin (FSK) because hESC-derived G-heporgs were not identical to PHH-derived heporgs. FSK activates aquaporin through cAMP/PKA signaling [34], inducing fluid influx that promoted the vesiculation and disrupted multipolar architecture (Fig. S8a, b). Additionally, FSK neither enhanced mature hepatocyte phenotypes nor reduced CK19 expression in G-heporgs (Fig. S8c). Although IL6 and OSM support the long-term expansion of PHH-derived heporgs [8, 17], they failed to improve proliferative capacity of G-heporgs (Fig. S9a, b). Importantly, GA017 withdrawal abolished G-heporg proliferation even in the presence of IL6 or OSM (Fig. S9a, b), and these cytokines minimally affected hepatocyte maturation-associated gene expression (Fig. S9c, d). Collectively, these findings underscore critical role of sustained YAP signaling in maintaining long-term G-heporg expansion and demonstrate that GA017 effectively preserves this proliferative capacity.

### Maturation of G-heporgs

**Fig. 5:**
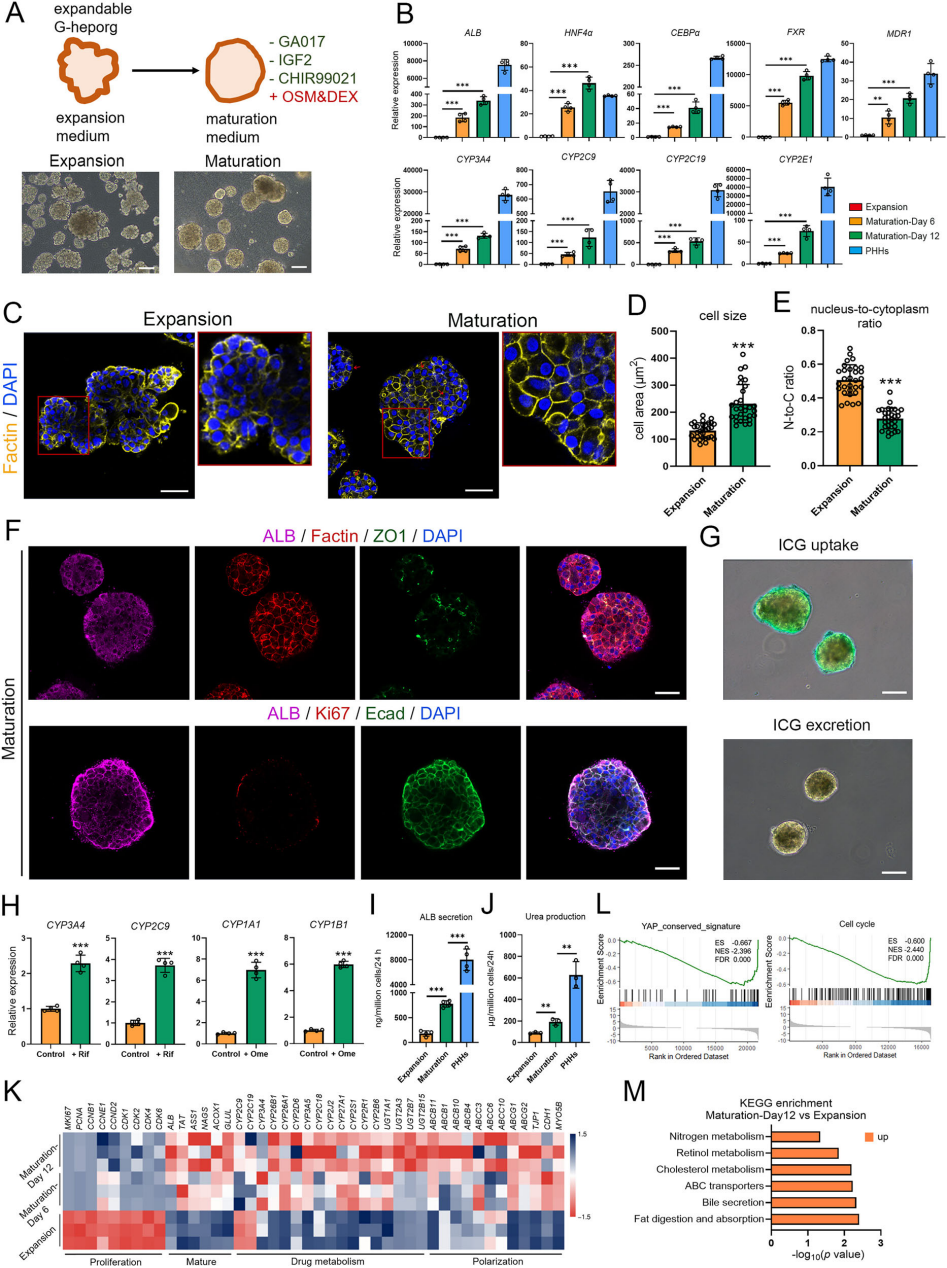
Differentiation of expandable G-heporgs into more mature hepatocyte organoid. **A** Schematic illustration of the differentiation of G-heporgs treated with GA017 into more mature hepatocyte organoids. Scale bar = 100 μm. **B** Dynamic gene expression of mature hepatocyte genes was analyzed by RT-qPCR at different stages and PHHs. *n* = 4 biologically independent experiments. **C** Representative Factin staining image of G-heporgs in expansion and maturation medium (12 days post switch). Red arrows indicated binucleated hepatocytes. Scale bar = 50 μm. **D**, **E** The quantification of cellular features of G-heporgs in expansion and maturation medium based on Factin (phalloidin) staining, including cell area (*n* = 30 cells) (**D**) and nucleus-to-cytoplasm ratio (*n* = 30 cells) (**E**). **F** Immunostaining of ALB, Factin, ZO1, Ki67, and E-cad. Nuclei were stained with DAPI. Scale bar = 50 μm. **G** Representative images of ICG uptake and excretion. Scale bar = 100 μm. **H** The expression of CYPs after treating with inducers was analyzed by RT-qPCR. *n* = 4 biologically independent experiments. Analysis for the secretion of ALB (*n* = 4 biologically independent experiments) (**I**) and the production of urea (*n* = 3 biologically independent experiments) (**J**). **K** Heatmap of G-heporgs in expansion and maturation medium for genes related to proliferation, mature, drug metabolism, and polarization. **L** GSEA analysis. **M** KEGG analysis. Results were presented as mean ± SD. Statistical significance was determined using one‑way ANOVA followed by Tukey post‑test and unpaired two-tailed Student’s *t* test. *p < 0.05, **p < 0.01, ***p < 0.001.

Although GA017-mediated YAP activation enabled stable long-term expansion of G-heporgs, it simultaneously suppressed hepatocyte maturation gene expression (Fig. 4G). To promote further maturation of expandable G-heporgs, we developed a defined maturation medium. In this medium, CHIR99021, IGF2, and GA017, which are factors essential for hepatocyte proliferation, were removed and replaced with Dexamethasone (Dex) and Oncostatin M (OSM) (Fig. 5A). RT-qPCR analysis showed significant upregulation of mature hepatocyte markers (*ALB*, *FXR*, *MDR1*, *CYPs*) and key hepatocyte transcription factors (*HNF4α*, *CEBPα*) (Fig. 5B). Mature organoids displayed characteristic polygonal hepatocyte morphology (Fig. 5C) with a significant increase in cell diameter (Fig. 5D), a reduced nuclear-to-cytoplasmic ratio (Fig. 5E), and a higher frequency of binucleated cells (indicated by red arrows) (Fig. 5C). IF assessment confirmed robust expression of mature hepatocyte marker ALB, along with epithelial marker Ecad and tight junction marker ZO1 at day 12 of maturation (Fig. 5F). Concurrently, loss of Ki67 expression indicated that cells exited the proliferative state following withdrawal of the three proliferative factors (Fig. 5F). Functional assays further demonstrated that mature G-heporgs efficiently performed indocyanine green (ICG) uptake and excretion, reflecting intact transport function (Fig. 5G). Treatment with cytochrome P450 (CYP) inducers rifampicin (Rif) and omeprazole (Ome) significantly increased expression of *CYP3A4*, *CYP2C9*, *CYP1A1*, and *CYP1B1* (Fig. 5H). Mature G-heporgs also exhibited enhanced ALB secretion (Fig. 5I) and urea synthesis (Fig. 5J). RNA-seq heatmap and GSEA analysis revealed pronounced downregulation of proliferation and YAP signaling genes alongside concurrent upregulation of maturation, drug metabolism, and polarization genes during the maturation (Fig. 5K, L). KEGG analysis of DEGs between mature (day 12) and expandable G-heporgs identified enrichment in hepatocyte functional pathways, including lipid metabolism, bile secretion, and cholesterol metabolism (Fig. 5M). Collectively, these findings demonstrated that our system efficiently induced further maturation of G-heporgs, although their functionality still fell short of that of PHHs (Fig. 5B, I, J).

### Characterization of multipolar architecture in expandable G-heporgs

**Fig. 6:**
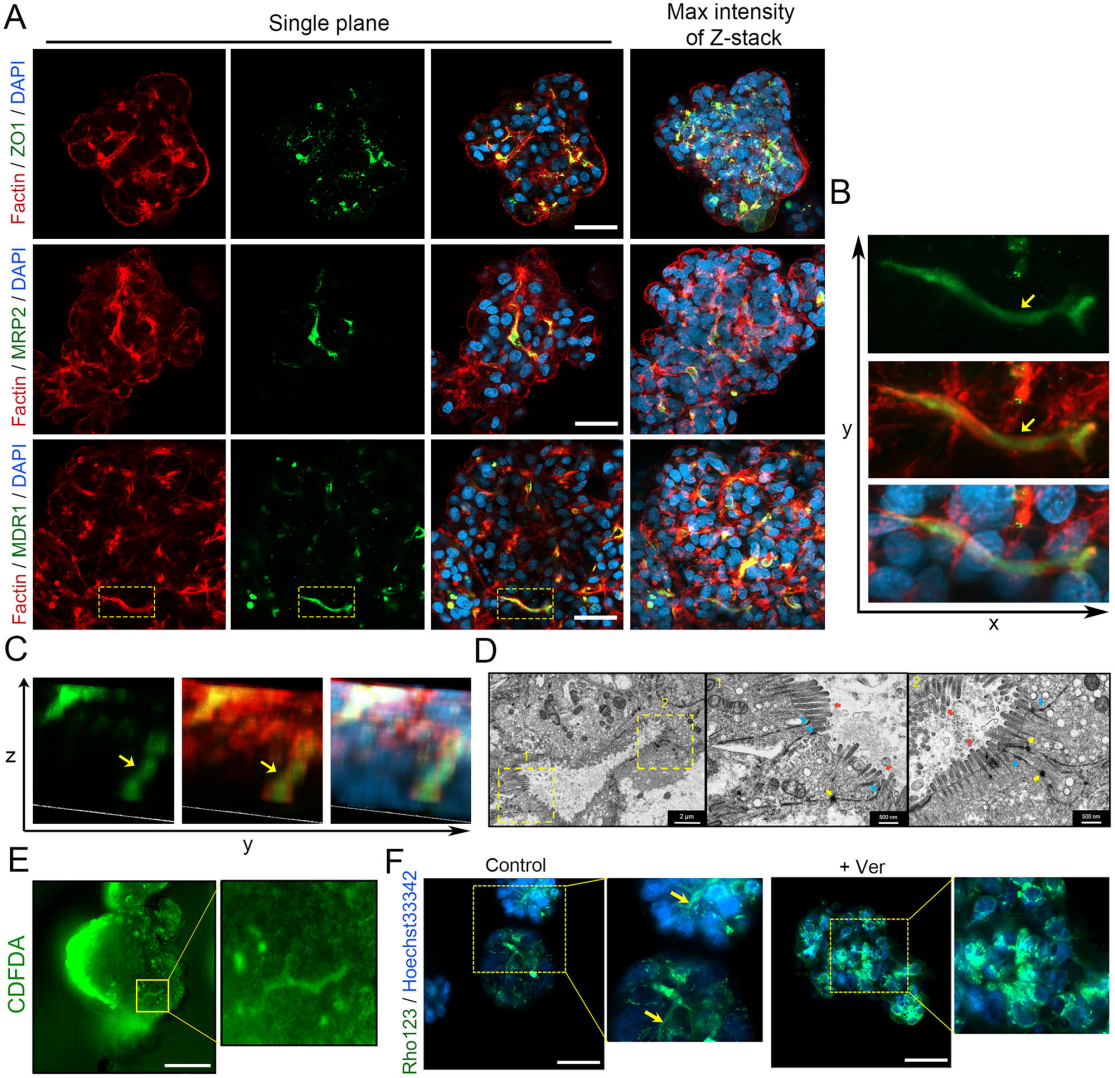
Characterization of the multi-polarization structure of expandable G-heporgs. **A** Immunostaining of Factin, ZO1, MRP2, and MDR1. Nuclei were stained with DAPI. Scale bar = 50 μm. **B**, **C** 3D imaging of MDR1 and Factin staining. **D** Representative images of transmission electron micrograph (TEM). Scale bar = 2 μm (left) and 500 nm (middle and right). Red arrows indicated microvilli, blue arrows indicated tight junction and yellow arrows indicated desmosomes. **E** Representative images of CDFDA staining. Scale bar = 100 μm. **F** Representative images of Rho123 staining with or without Ver (Verapamil) treatment. Scale bar = 50 μm.

Expandable G-heporgs maintained multipolar architecture during long-term culture. IF staining for ZO1 and the apical hepatocyte membrane markers Multidrug Resistance-Associated Protein 2 (MRP2) and MDR1 confirmed that expandable G-heporgs preserved the capacity to form BC networks (Fig. 6A), consistent with in vivo hepatocyte organization [11, 35]. Three-dimensional imaging further verified laterally oriented MDR1-positive BC (indicated by yellow arrows) (Fig. 6B, C). Transmission electron microscopy (TEM) revealed the formation of BC with characteristic microvilli and adjacent tight junctions (Fig. 6D). In live cells of expandable G-heporgs, the fluorescent substrates 5(6)-carboxy-2’,7’-dichlorofluorescein diacetate (CDFDA; for MRP2 activity test) and Rhodamine 123 (Rho123; for MDR1 activity test) were actively secreted and accumulated in luminal spaces (Fig. 6E, F), demonstrating intact transporter functionality and luminal integrity. These findings established that expandable G-heporgs developed apical-basolateral polarity and functional bile canaliculi.

### Expandable G-heporgs recapitulate cholestasis and regulate polarized trafficking

**Fig. 7:**
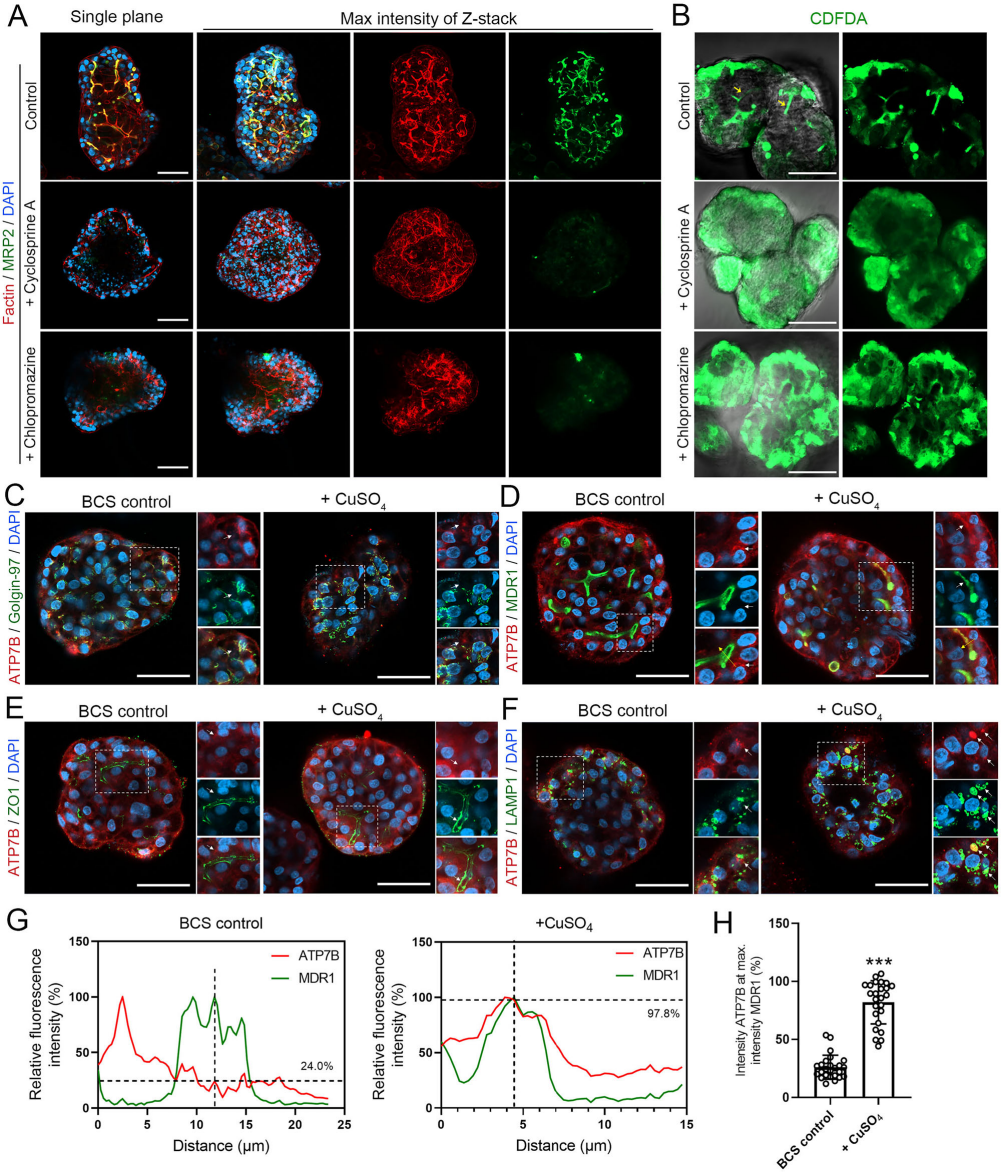
Expandable G-heporgs recapitulated copper-stimulated ATP7B translocation. **A** Immunofluorescence staining of F-actin and MRP2 in expandable G-heporgs under the indicated conditions. Nuclei were stained with DAPI. Scale bar = 50 μm. **B** Representative images of CDFDA staining in expandable G-heporgs under the indicated conditions, showing canalicular bile acid transport activity. Scale bar = 50 μm. Immunostaining of ATP7B and Goligin-97 (**C**) ATP7B and MDR1 (**D**) ATP7B and ZO1 (**E**) ATP7B and LAMP1 (**F**) in BCS-treated and CuSO4-treated expandable G-heporgs. Nuclei were stained with DAPI. Scale bar = 50 μm. White arrows indicated ATP7B colocalization with or without the above markers, and yellow arrows indicated the plot quantification direction. **G** Fluorescence intensity profile plots along the yellow arrows showing overlapping of ATP7B and MDR1 intensity peaks in G-heporgd treated with CuSO4, but not in the BCS-control group. **H** The percentage values of ATP7B intensity at which MDR1 intensity was at its maximum (*n* = 25 cells). Results were presented as mean ± SD. Statistical significance was determined using unpaired two-tailed Student’s t-test. ***p < 0.001.

In vitro hepatocyte culture models that faithfully recapitulate the bile canalicular network are crucial for establishing disease models and predicting drug toxicity, particularly for compounds that cause cholestasis [36]. Previously, only sandwich hepatocyte culture models could reproduce bile canalicular structures resembling those found in vivo, but their scalability remains limited [37]. To evaluate whether our expandable G-heporgs could serve as functional in vitro hepatocyte organoid models with bile canalicular networks, we examined their responses to known cholestasis-inducing drugs, Cyclosporine A and Chlorpromazine. Upon the treatment, IF analysis revealed that both drugs markedly reduced MRP2 expression and disrupted its proper localization to the bile canaliculi, whereas the control group displayed clear and highly branched MRP2-positive bile canalicular networks (Fig. 7A). Furthermore, using a functional CDFDA-based bile canaliculi assay, we observed that fluorescent CDF was efficiently secreted into the bile canalicular in control organoids, whereas drug treatment led to intracellular retention of CDF (Fig. 7B). These results demonstrated that our expandable G-heporgs not only formed functional bile canalicular networks but also faithfully recapitulated the adverse effects of cholestasis-inducing drugs on the bile canalicular networks.

In addition, to investigate whether expandable G-heporgs could model polarized trafficking, we examined the transportation of copper ions. Under physiological conditions, the copper transporter ATP7B localizes to the trans-Golgi network (TGN), however, in response to elevated copper levels, ATP7B undergoes polarized translocation to BC domain to facilitate the excretion of excess copper [38, 39]. In expandable G-heporgs, ATP7B co-localized with TGN marker Golgin-97 (Fig. 7C, left panel), but was absent from MDR1- and ZO1-labeled BC (Fig. 7D, E, left panel). Upon copper treatment, ATP7B co-localization with Golgin-97 decreased (Fig. 7C, right panel), accompanied by redistribution to BC domains bordered by MDR1 and ZO1 (Fig. 7D, E, right panel). Furthermore, copper exposure enhanced ATP7B co-localization with lysosomal-associated membrane protein 1 (LAMP1) (Fig. 7F, right panel), whereas minimal overlap was observed in untreated cells (Fig. 7F, left panel), consistent with previous evidence that LAMP1 mediates ATP7B trafficking to bile canaliculi [40]. Fluorescence intensity profiles across ATP7B and BC domains demonstrated translocation, reflected by overlapping MDR1 and ATP7B peaks under copper-treated conditions compared with bathocuproinedisulfonic acid disodium salt (BCS)-treated controls (Fig. 7G). Quantification of relative ATP7B intensity at MDR1 maxima across multiple BC revealed significantly increased ATP7B staining in copper-treated expandable G-heporgs compared with BCS-treated controls (Fig. 7H). These findings demonstrated that expandable G-heporgs not only established apical-basolateral polarity and functional BC but also faithfully recapitulated regulated polarized trafficking. This property highlights their utility for modeling liver diseases involving polarized trafficking defects (e.g., Wilson disease) and other disorders affecting specialized transport processes.

## Discussion

The advances in heporg technology provide a novel and more physiologically relevant platform for studying fundamental liver biology and modeling hepatic diseases [9, 10]. However, current approaches rely exclusively on PHHs, limiting broader application due to donor variability and restricted tissue availability. In this study, we successfully generated hESC-derived G-heporgs that recapitulated key features of PHH-derived heporgs, including ALB expression, proliferative capacity, and the formation of functional BC network.

Unlike the typical columnar polarization of most epithelial cells, hepatocytes exhibit unique multipolar organization, with one or more apical domains forming tubular bile canaliculi between adjacent hepatocytes. These canaliculi create a continuous network that drains bile into cholangiocyte-lined ductules and ultimately into the bile ducts [41]. This specialized polarity enables hepatocytes to simultaneously perform basolateral uptake of metabolites from blood and apical excretion of toxins into bile, thereby maintaining essential detoxification and metabolic functions. By contrast, organoids derived from cholangiocytes or liver progenitor cells generally form cystic structures with columnar polarization, thus fail to establish interconnected, functional bile canaliculi networks [7, 15]. Although the conventional sandwich culture model can partially recapitulate the multipolar architecture of hepatocytes, it does not reproduce their native three-dimensional organization [42]. Therefore, our expandable G-heporgs with functional BC networks cultured under 3D conditions more closely mimic in vivo polarized trafficking processes.

Building on the establishment of multipolar hepatocyte architecture and functional bile canalicular networks, expandable G-heporgs provide a versatile platform for studying hepatocyte-specific polarized trafficking and its disruption in disease contexts. The preserved responsiveness of canalicular transporters to cholestasis-inducing drugs, together with intact vectorial secretion into bile canaliculi, enables mechanistic investigation of cholestatic drug-induced liver injury (DILI), a major challenge in current preclinical toxicology [43]. In contrast to conventional hepatocyte culture systems that rapidly lose polarity, G-heporgs maintain stable multipolar architecture and interconnected bile canaliculi, thereby offering a physiologically relevant model for predicting cholestatic DILI risk and dissecting transporter-mediated drug toxicity. Moreover, the regulated copper-dependent redistribution of ATP7B highlights the suitability of this system for modeling inherited disorders of polarized trafficking, such as Wilson disease, as well as other liver diseases associated with BC dysfunction [44]. In this context, G-heporgs derived from patient-specific iPSCs may further facilitate personalized disease modeling and drug screening, particularly for conditions in which bile canalicular dysfunction and impaired transporter trafficking represent central pathogenic mechanisms.

In contrast to hepatic progenitor/cholangiocyte organoid cultures, the generation and long-term expansion of grape-like clusters of mature hepatocyte-derived organoids depend heavily on liver regeneration signaling. Previous studies have shown that inflammatory cytokines such as TNFα and IL6 are critical for sustaining the expansion of hepatocyte organoids derived from both mouse and human primary hepatocytes [9, 10, 17]. More recent findings demonstrated that Wnt ligands, OSM-STAT3 signaling, and bile acid-FXR signaling markedly enhance the prolonged propagation of hepatocyte organoids generated from mouse and human primary hepatocytes, as well as human iPSC-derived hepatocytes [8, 11, 45]. These pathways are well-established regulators of liver regeneration [5, 46]. In our study, an inflammatory microenvironment induced by S-heporgs promoted IGF2 production in G-heporgs, thereby supporting G-heporg formation and expansion. However, due to the complexity of S-CM, we have not yet identified the specific inflammatory factor(s) driving IGF2 induction. Future studies aimed at dissecting these signals may not only clarify the mechanism underlying IGF2 regulation but also enable further optimization of culture conditions to enhance G-heporg expansion. Additionally, we also showed that the YAP signaling robustly facilitated their long-term propagation. Both IGF2 and YAP signaling are indispensable mechanisms in liver regeneration. As a crucial mitogen in this process [47], IGF2 further enhanced the expansion of G-heporgs by activating the PI3K-AKT signaling pathway. The overexpression of YAP signaling rapidly induces liver enlargement [48] and promotes the dedifferentiation of mature hepatocytes into progenitor-like cells [21], whereas reduced YAP activity favors hepatocyte maturation [49]. Accordingly, combined treatment with GA017 (YAP agonist) and IGF2 enabled sustained expansion of G-heporgs for more than two months while preserving structural integrity. The withdrawal of these proliferative stimuli subsequently promoted the acquisition of mature functional phenotypes. Together, these findings indicated that our G-heporg culture system effectively recapitulated key aspects of in vivo hepatocyte regeneration, supporting the generation of regeneration-competent G-heporgs.

Although our differentiated G-heporgs maintained long-term expansion and recapitulated hepatocyte-specific polarized architectures of the liver, their fundamental limitation lies in their exclusively hepatocyte composition. Non-parenchymal cells (NPCs) in the liver, including hepatic stellate cells, Kupffer cells, and liver sinusoidal endothelial cells, also play essential roles in hepatocyte development and disease pathogenesis [50]. Moreover, unlike PHH-derived heporgs, G-heporgs showed minimal proliferation in response to IL-6 or OSM [8, 17]. We attributed this distinction to their origin: G-heporgs were generated from hESC-derived HB-orgs [15], whereas PHH-derived heporgs were produced directly from fully mature hepatocytes isolated from PHHs [9]. Despite progressive improvements in functional maturity of hESC-derived heporgs, notable differences persist compared with PHHs [51].

Notably, differentiated G-heporgs no longer expressed classical cholangiocyte or progenitor markers such as SOX9 and TBX3. However, they consistently retained CK19 expression, regardless of YAP modulation. By contrast, PHH-derived heporgs are typically CK19-negative under basal conditions and upregulate CK19 only upon YAP activation [8, 17]. The persistent CK19 expression observed in G-heporgs, therefore, likely reflects a distinct cellular identity rather than an induced progenitor state. In line with this notion, transcriptomic comparisons revealed that G-heporgs shared molecular features with fetal hepatocytes (Fig. S7), suggesting that these cells reside in a fetal-like hepatocyte state rather than a fully mature adult hepatocyte identity. Such a state may confer enhanced cellular plasticity and long-term expandability, but may also contribute to functional differences relative to PHH-derived heporgs, including reduced albumin secretion and attenuated hepatocyte-specific metabolic functions. Future efforts integrating non-parenchymal cell co-culture systems with further optimization of in vitro maturation strategies may enable the generation of more complex, multicellular hepatocyte organoids that more closely approximate the functional maturity and physiological heterogeneity of the native human liver [11, 52, 53].

In summary, by co-modulating YAP and IGF2 signaling, we established a defined culture system to generate hESC-derived expandable grape-clustered hepatocyte organoids with functional bile canalicular networks. These organoids closely mimic the architecture of in vivo hepatocytes, providing a valuable platform for investigating polarized metabolite trafficking and modeling liver diseases.

## Materials and methods

### Differentiation and culture of organoid from hESCs

The hESC lines, H9 cells, were obtained from WiCell Research Institute (Madison, WI, USA) under Materials Transfer Agreements (No. 19-W0512, 24-W0162, 24-W0163). The culture of H9 cells was described previously [15]. In brief, H9 cells were cultured on mouse embryonic fibroblast feeder layers in DMEM/F12 medium (Gibco, C11330500BT), containing 20% knockout serum replacement (KSR, Gibco, 10828028), 1% non-essential amino acids (NEAA, Gibco, 11140050), 0.1 mM 2-mercaptoethanol (Sigma, M3148), 1% GlutaMax I (Gibco, 35050061) and 10 ng/mL bFGF (PeproTech, 100-18B), and maintained in a humidified incubator at 37 °C and 5% CO_2_. The culture medium was changed daily. The differentiation and culture of HB-org were described previously [15]. For the differentiation and culture of G-heporgs, HB-orgs were harvested and washed with PBS, followed by dissociation into single cells using TrypLE. A total of 2 × 10^5^ cells were then reseeded in heporg medium composed of IMDM supplemented with 10% FBS, 1% NEAA, 1% GlutaMax I, 1% Penicillin-Streptomycin, 2.5 mM Nicotinamide (Sigma, N0636), 3 μM CHIR99021 (MedChemExpress, HY-10182), 10 μM SB431542 (MedChemExpress, HY-10431), 10 μM Y27632 (MedChemExpress, HY-10071), 50 ng/mL FGF4 (PeproTech, 100-31) and 50 ng/mL EGF (PeproTech, AF-100-15), supplemented with dispersing 5% growth factor reduced Matrigel (Corning, 354230) (only added when passage) and maintained in a humidified incubator at 37 °C and 5% CO_2_. The medium was changed daily and organoids were passaged after 5–7 days at a ratio of 1:3. On the 7–14 days of the third generation, the representative grape-like organoids would be observed. During subsequent optimization, 25 ng/mL IGF2 (Peprotech, 100-10) was identified as a key factor promoting G-heporg formation and was thus incorporated into the final optimized heporg medium (OHM). For continuous expansion of G-heporgs (expandable G-heporgs), the G-heporg expansion medium (EM) was prepared based on the optimized heporg medium described above, with the addition of 10 μM GA017 (MedChemExpress, HY-147082). To test the effect of inflammatory factors, cytokines and inhibitors, 50 ng/mL TNFα (Peprotech, 300-01 A), 20 ng/mL TGFα (Peprotech, 100-16 A), 20 ng/mL IL10 (Peprotech, 200-10), 20 ng/mL IL11 (Peprotech, 200-11), 100 ng/mL IL6 (Peprotech, 200-06), 20 ng/mL Oncostatin M (OSM, Peprotech, 300-10), 10 μM LY294002 (MedChemExpress, HY-10108), 1 μM Linsitinib (MedChemExpress, HY-10191) and 1 μM Verteporfin (MedChemExpress, HY-B0146) was added into the EM respectively.

To quantify G-heporg formation efficiency, dissociated single cells were seeded at a density of 10,000 cells per well into ultra-low-attachment 48-well plates and cultured under the indicated conditions. At the end of the culture, the total number of G-heporgs formed in each well was counted under a bright-field microscope. Formation efficiency was calculated and expressed as the number of G-heporgs formed per 10,000 input cells.

To test the function of S-heporg-conditioned medium (S-CM), culture medium from separately cultured S-heporgs was collected daily and centrifuged at 900 × *g* for 10 min to remove cellular debris. The clarified medium was then filtered through a 0.22 µm syringe filter to remove any remaining cells and stored at −20 °C until use. For functional assays, S-CM was mixed with fresh culture medium at defined ratios to achieve final concentrations of 25% and 50% (v/v).

For the maturation, G-heporgs were collected and transferred into maturation medium, which was composed of EM but without GA017, IGF2, CHIR99021 and with the addition of 20 ng/mL OSM and 200 nM dexamethasone (Dex, Sigma, D4902), supplemented with dispersing 5% growth factor reduced Matrigel and maintained in a humidified incubator at 37 °C and 5% CO_2_. The medium was changed daily.

### Culture of PHHs

PHHs were isolated from excess liver tissue obtained from patients undergoing surgical liver resection. The use of human liver tissue was approved by the Research Ethics Committee of Guangzhou First People’s Hospital (Ethical Approval No. K-2019-167-02). Hepatocytes were isolated using a modified two-step collagenase perfusion procedure. The isolated PHHs were seeded onto type I collagen (Corning, C0130) coated 6-well plates. Cells were cultured in hepatocyte culture medium (HCM; Lonza, CC-3199) supplemented with SingleQuots (Lonza, CC-4182) minus EGF, 1% B27 supplement (Gibco, 17504044), 100 nM Dex, 20 ng/mL FGF4, 20 ng/mL hepatocyte growth factor (HGF, PeproTech, 100-39), and 40 ng/mL oncostatin M (PeproTech, 300-10), when indicated.

### Reverse transcription quantitative polymerase chain reaction (RT‑qPCR)

Total RNAs were extracted using the RNAiso Plus kit (Takara, 9109) according to the manufacturer’s instructions. One microgram of RNA was reverse transcribed into cDNA using the HiScript III RT SuperMix for qPCR (Vazyme, R323-01). RT-qPCR was performed using ChamQ Universal SYBR qPCR Master Mix (Vazyme, Q711-03) and 96-well qPCR plate (MIKX, MK1009) on the QuantStudio 1 Real-Time PCR system (ABI, Thermo, USA). The cycle threshold (CT) values for each sample were normalized to the expression of the housekeeping gene glyceraldehyde-3-phosphate dehydrogenase (GAPDH). Relative gene expression levels were calculated using the 2^−ΔΔCT^ method. The primer sequences used for RT-qPCR are listed in Supplementary Table 1.

### Immunofluorescence (IF) staining

Organoids were collected at designated time points, washed with PBS, and fixed overnight at 4 °C in 4% paraformaldehyde (PFA). Following the fixation, organoids were permeabilized with 0.5% Triton X-100 for 20 min and blocked with goat or donkey serum for 60 min. After each step, organoids were washed three times with PBS. Organoids were then incubated with primary antibodies diluted in PBS overnight at 4 °C, followed by the incubation with secondary antibodies in PBS for 1 h at room temperature in dark. Nuclei were counterstained with DAPI (Beyotime Biotechnology, C1006) for 5 min. Immunostaining images were captured using a single-photon confocal microscope (LSM 900, Zeiss, Germany). The antibodies used in this study were listed in Supplementary Table 2.

### Western blot (WB) analysis

Cells were lysed on ice using RIPA lysis buffer (Solarbio, R0020) supplemented with PMSF (Beyotime Biotechnology, ST505) and protease inhibitor (Beyotime Biotechnology, P1005). Protein concentrations were determined using the Bicinchoninic Acid (BCA) Protein Assay Kit (Biosharp, BL521A) following the manufacturer’s instructions. Western blotting was performed using standard procedures. The antibodies used in this study were listed in Supplementary Table 2. The original full and uncropped Western blots could be found in Supplementary Material.

### Analysis for liver function

To analyze the secretion levels of ALB, medium was collected at indicated time points and analyzed by using Human Albumin ELISA Quantitation Kit (Bethyl, E88-129) following manufacturer’s instructions. To analyze the production of urea, 10 mM ammonium chloride (Sigma, A9434) was added into the culture medium and incubated for 24 h, then the medium was collected and Urea Assay Kit (Solarbio, BC1535) was used for the quantification according the manufacturer’s protocols. For the assessment of the uptake and excretion of indocyanine green (ICG; MedChemExpress, HY-D0711), organoids were incubated with 1 mg/mL ICG for 1 h at 37 °C under 5% CO₂ to assess uptake. Following the incubation, ICG uptake was visualized under a microscope, after the organoids were gently washed three times with PBS to remove residual dye. Fresh culture medium was then added, and ICG excretion was monitored by microscopy 1 h later to evaluate functional clearance.

### Lipid droplet staining

Organoids were washed by PBS and fixed with 4% PFA for 20 min at room temperature. After washed by PBS three times, organoids were stained with BODIPY 493/503 (GLPBIO, GC42959) solution for 30 min. After washed by PBS three times, nuclei were counterstained with DAPI for 5 min. The fluorescent images were captured using a single-photon confocal microscope. The relative lipid area was quantitative by using ImageJ software.

### Senescence-associated β-galactosidase (SA-β-gal) staining

SA-β-gal staining was performed using the SA-β-gal Staining Kit (Beyotime Biotechnology, C0602) following the manufacturer’s instructions. The organoids were first fixed with 4% PFA for 30 min at room temperature, followed by overnight incubation in X-gal solution at 37 °C in the dark. After staining, the organoids were washed with PBS and imaged under a phase-contrast microscope (Nikon, Japan) with random field selection.

### TdT-mediated dUTP nick-end labeling (TUNEL) assay

TUNEL assay was performed using One-Step TUNEL Apoptosis Assay Kit (Beyotime Biotechnology, C1086) following the manufacturer’s protocol. Briefly, organoids were fixed with 4% PFA for 30 min and permeabilized with 0.5% Triton X-100 for 20 min at room temperature. Subsequently, the samples were incubated with TUNEL detection mixture for 1 h in the dark. Nuclei were then counterstained with DAPI for 5 min. After washing with PBS, the organoids were randomly imaged using single-photon confocal microscopy.

### Enzyme-Linked Immunosorbent Assay (ELISA)

During the differentiation and culture of G-heporgs, culture supernatants were collected at the indicated time points. The concentrations of IGF2, IL32, and CCL20 were quantified using a Human IGF2 ELISA kit (Jingmei Biotechnology, JM-0862H2), a Human IL32 ELISA kit (SAB), and a Human CCL20 ELISA kit (Jingmei Biotechnology, JM-5919H2), respectively, according to the manufacturers’ instructions.

### Transmission electron microscopy (TEM)

Organoids were fixed overnight at 4 °C in 2.5% glutaraldehyde, followed by post-fixation in 1% osmium tetroxide for 2 h. After the dehydration through a graded ethanol series, the samples were embedded, sectioned using Leica UC7 ultramicrotome, and sequentially stained with 2% uranyl acetate (20 min) and lead citrate (12 min). Finally, the ultrathin sections were imaged using an FEI transmission electron microscope (Tecnai G2 Spirit, USA).

### Karyotyping

For karyotyping, organoids in the active growth phase at passage 10 were treated with 0.4 μg/mL colcemid for 3 h at 37 °C to arrest cells in metaphase. Organoids were then dissociated into single cells using TrypLE. Following centrifugation and removal of the supernatant, cells were incubated in 0.075 M KCl for 30 min at 37 °C and subsequently fixed in a 3:1 methanol-acetic acid solution. After fixation, the cells were rinsed three times with fresh fixative prior to slide preparation. G-banded karyotype analysis was performed according to standard protocols.

### Bile canaliculi assessment

For bile canaliculi assessment, organoids were incubated with 2 μM 5(6)-carboxy-2’, 7’-dichlorofluorescein diacetate (CDFDA, sigma, 21884) at 37 °C for 15 min, followed by three times PBS washes. Fluorescence imaging was performed immediately using single-photon confocal microscopy.

### Rhodamine transport assay

G-heporgs were incubated with 100 μM rhodamine 123 (MedChemExpress, HY-D0816) at 37 °C for 30 min. To confirm that rhodamine 123 efflux specifically reflected multidrug resistance protein 1 (MDR1) activity, parallel experiments were performed following pretreatment with 10 μM verapamil (MedChemExpress, HY-14275) at 37 °C for 30 min, after which the rhodamine uptake assay was repeated.

### Cholestasis-inducing drug treatment

Expandable G-heporgs were treated with Cyclosporine A (10 µM, MedChemExpress, HY-B0579) or Chlorpromazine (1 µM, Selleck, S5749), respectively. Control groups were cultured in parallel with 0.1% DMSO treatment. After 48 h, organoids were collected and the BC integrity was detected.

### Copper metabolism

To assess copper metabolism and ATP7B translocation, G-heporgs were treated with 200 μM bathocuproine disulfonic acid disodium salt (BCS, Aladdin, B486593) for 8 h, washed with PBS, and then incubated with either 200 μM BCS or 100 μM CuSO₄ (MedChemExpress, HY-Y1881B) for 16 h, then organoids were collected, washed with PBS for three times and fix in 4% PFA for subsequent IF staining analysis.

### RNA-seq analysis

Total RNAs of organoids were extracted using RNAiso Plus kit according to the manufacturer’s instructions. The cDNA libraries for RNA sequencing were generated using NEBNext® Ultra™ RNA Library Prep Kit for Illumina (NEB, E7530). Sequencing was performed by Novogene (Beijing, China) on an Illumina HiSeq X-Tensequencer with 150 bp paired-end sequencing reaction. Differentially expressed genes (DEGs) were analyzed by DESeq2 using counts. Genes with *P* value ≤ 0.05 and fold change ≥2 were identified as DEGs. Original data were uploaded to the Gene Expression Omnibus database (accession number: GSE302458). The ligand-receptor pairings were searched using the CellTalkDB database [27].

### Statistical analysis

Sample sizes were indicated on the corresponding graph or figure legend; otherwise, *n* = 3, which represented the number of biological replicates that were analyzed in each experimental group. Data were expressed as the mean ± standard deviation. Statistical analysis was performed using SPSS. The unpaired, two-tailed Student’s *t* test, one‑way ANOVA followed by Tukey post‑test and two-way repeated-measures ANOVA followed by Tukey’s multiple comparisons test were used to evaluate statistical significance. Differences were considered statistically significant at *p*-value < 0.05.

## Supplementary information

Supplementary Information

Original WB images

## Data Availability

The data that support the findings of this study are available from the corresponding author upon request. The RNA-seq datasets have been deposited in GEO with accession number GSE302458.
