# Supplementary material for: Generation of proliferative hESC-derived grape-clustered hepatocyte organoids with multipolar architecture as regenerative counterpart via synergy of YAP and IGF2 pathways
Source: Cell Death Dis. 2026 Mar 26;17(1):381. doi: 10.1038/s41419-026-08635-y (PMC13039810; doi:10.1038/s41419-026-08635-y)
Supplement: Supplementary file 1 — Supplementary Information [file 41419_2026_8635_MOESM1_ESM.docx]

Supporting Information

**Generation of proliferative hESC-derived grape-clustered hepatocyte organoids with multipolar architecture as regenerative counterpart via synergy of YAP and IGF2 pathways**

Haibin Wu^1,2,3^, Shoupei Liu^1,2,3^, Sen Chen^1,2,3^, Changlu Qin^1,2,3^, Wenjiao Yan^1,2,3^, Xiangting Cao^1,2,3^, Yongjian Zhou^1^*, Yuyou Duan^1,2,3,4,5^*

**Affiliations:**

^1^Department of Gastroenterology and Hepatology, Guangzhou Digestive Disease Center, the Second Affiliated Hospital, School of Medicine, South China University of Technology, Guangzhou, 510006, China.

^2^Laboratory of Stem Cells and Translational Medicine, Center for Medical Research on Innovation and Translation, Institute of Clinical Medicine, the Second Affiliated Hospital, School of Medicine, South China University of Technology; Guangzhou, 510006, China.

^3^Laboratory of Stem Cells and Translational Medicine, Institute for Life Science, School of Medicine, South China University of Technology; Guangzhou, 510006, China.

^4^National Engineering Research Center for Tissue Restoration and Reconstruction, South China University of Technology; Guangzhou, 510006, China.

^5^The Innovation Centre of Ministry of Education for Development and Diseases, the Second Affiliated Hospital, School of Medicine, South China University of Technology; Guangzhou 510006, China.

*Corresponding author: [yuyouduan@scut.edu.cn](mailto:yuyouduan@scut.edu.cn), [yongjian_zhou@outlook.com](mailto:yongjian_zhou@outlook.com)


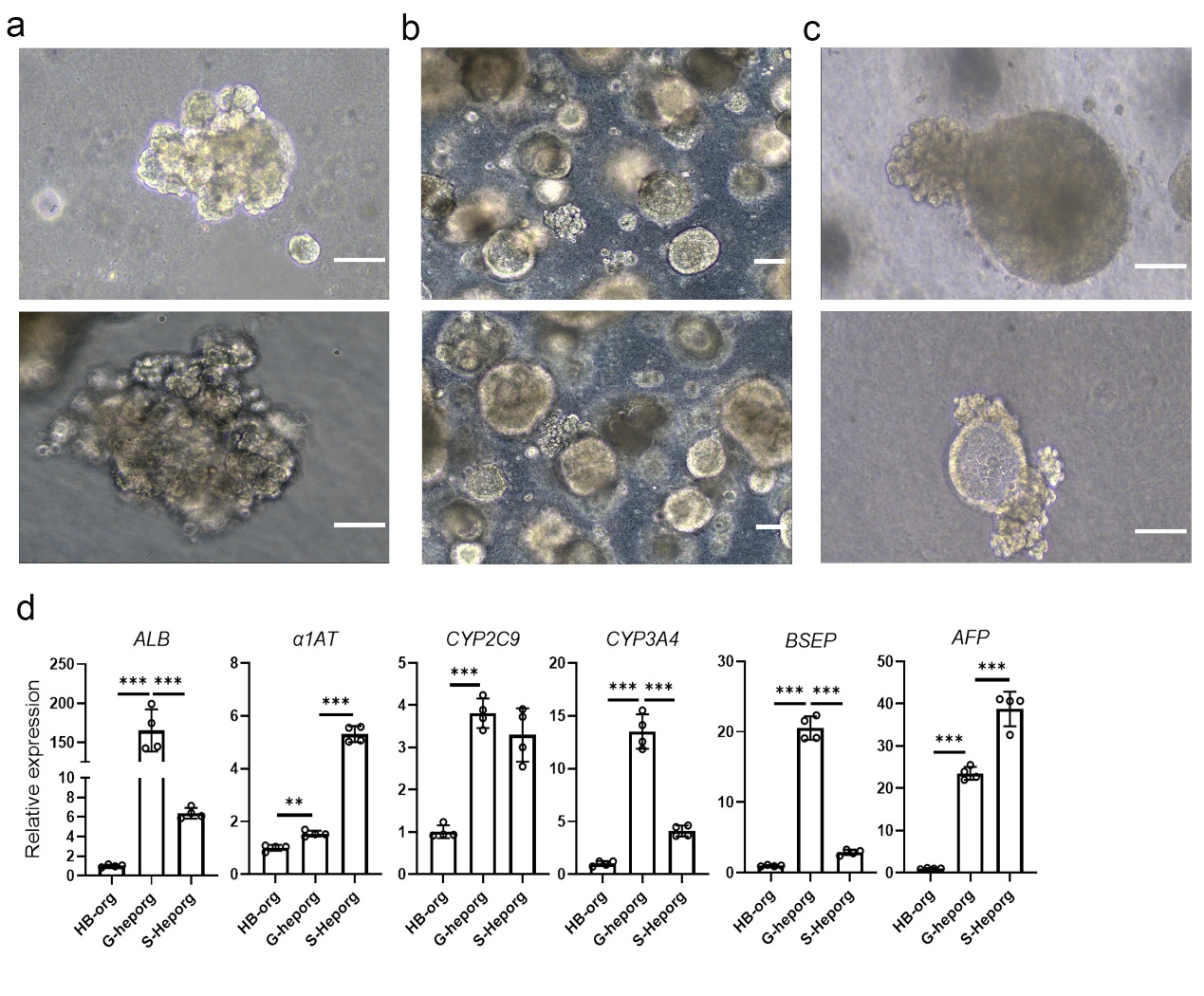


**Fig. S1 The morphologies and gene expressions of G-heporgs**

(a) Representative images of G-heporgs. Scale bar = 100 μm. (b) Representative images of G-heporgs and S-heporgs. Scale bar = 100 μm. (c) Representative images of G-heporgs which grown from S-heporgs. Scale bar = 100 μm. (d) The expression of hepatocyte related genes was analyzed by RT-qPCR in indicated organoids. n = 4 biologically independent experiments. Results were presented as mean ± SD. Statistical significance was determined using one‑way ANOVA followed by Tukey post‑test. ****p* <0.001.


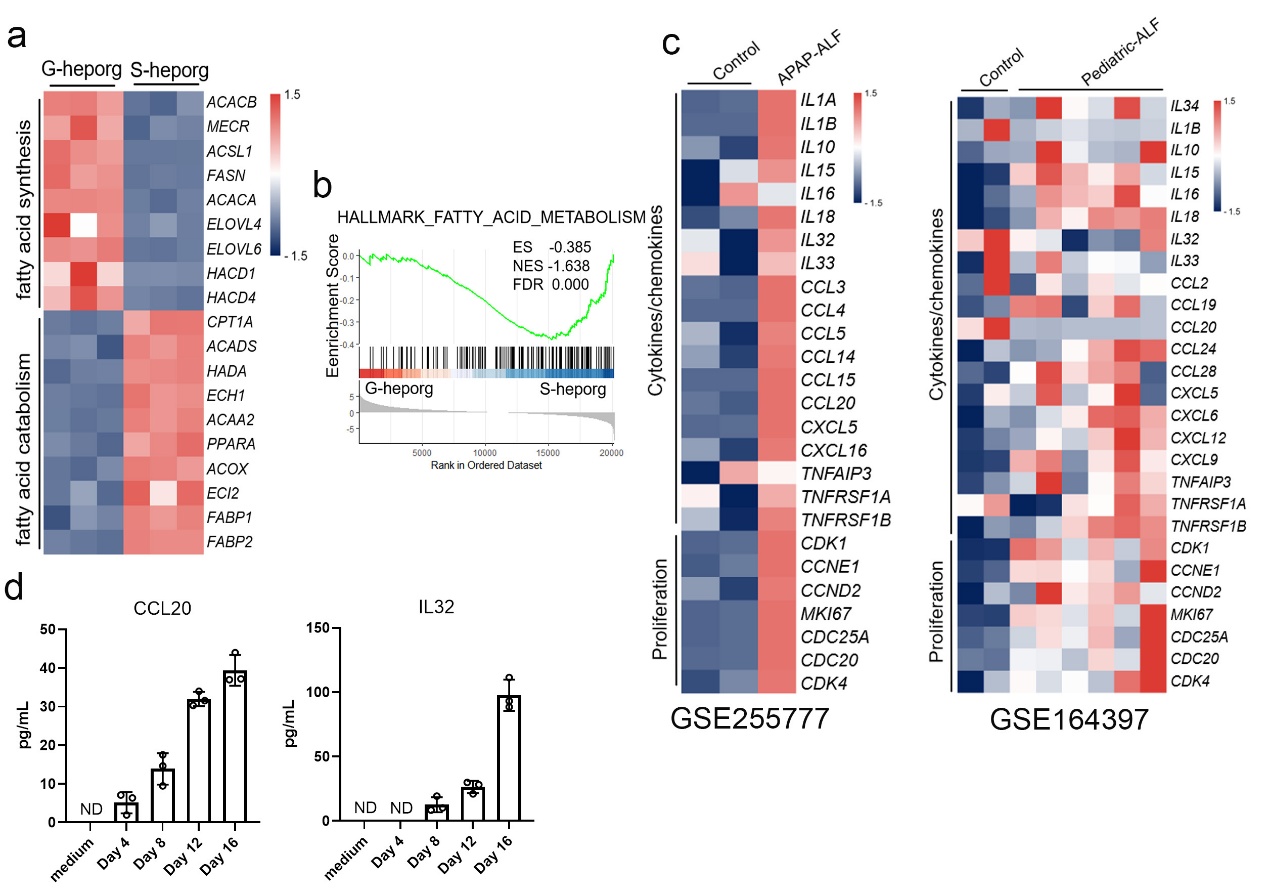


**Fig. S2 RNA-seq analysis of G-heporgs and S-heporgs**

(a) Heatmap of G-heporgs and S-heporgs for genes related to fatty acid synthesis and catabolism. (b) GSEA analysis. (c) Heatmap of normal and APAP-acute liver failure (ALF) (left) and Pediatric-ALF (right) for genes related to cytokines/chemokines and proliferation. (d) CCL20 and IL32 secretion from culture medium at different days. ND, not detected. n = 3 biologically independent experiments. Results were presented as mean ± SD.


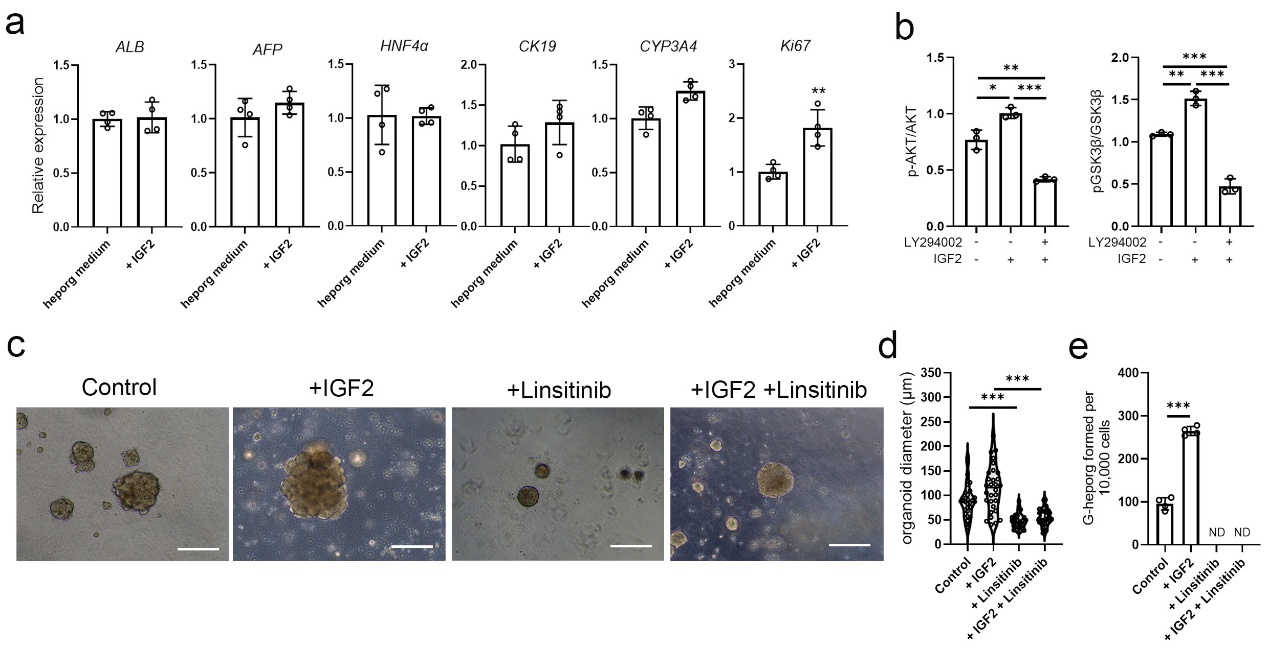


**Fig. S3 IGF2 was important for the generation and expansion of G-heporgs**

(a) The expression of hepatocyte related genes was analyzed by RT-qPCR in G-heporgs treated with or without IGF2. n = 4 biologically independent experiments. (b) Densitometric quantification of protein expression from Western blot analysis shown in Fig. 3K. n = 3 biologically independent experiments. (c) Representative images of G-heporgs after the treatment with or without IGF2 and Linsitinib. Scale bar = 100 μm. (d, e) Quantification of the diameter (n = 30 organoids) (d) and formation efficiency (n = 4 biologically independent experiments) (e) of G-heporgs at day 14 after different treatments. ND, not detected. Results were presented as mean ± SD. Statistical significance was determined using one‑way ANOVA followed by Tukey post‑test and unpaired two-tailed Student’s t-test. **p* <0.05, ***p* <0.01, ****p* <0.001.


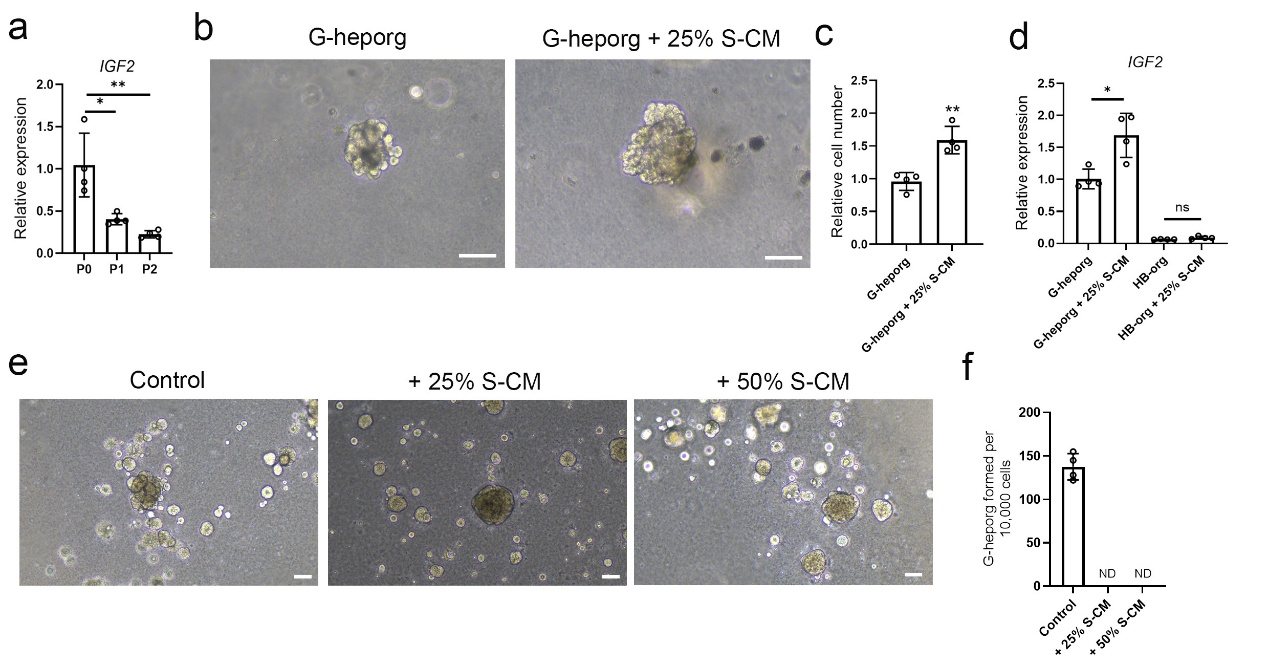


**Fig. S4 IGF2 was induced by S-heporg-conditioned medium (S-CM)**

(a) The expression of IGF2 was analyzed by RT-qPCR in G-heporgs after the separation from S-heporgs and subsequent independent culture. n = 4 biologically independent experiments. (b) Representative images of G-heporgs cultured in the presence or absence of 25% S-CM for 7 days. Scale bar = 100 μm. (c) Quantification of relative cell number of G-heporgs cultured with or without 25% S-CM for 7 days. n = 4 biologically independent experiments. (d) The expression of IGF2 was analyzed by RT-qPCR in indicated groups. n = 4 biologically independent experiments. (e) Representative images of HB-orgs cultured in the presence or absence of 25% S-CM throughout the differentiation into G-heporgs. Scale bar = 100 μm. (f) Quantification of G-heporg formation efficiency under the indicated conditions. ND., not detected. n = 4 biologically independent experiments. Results were presented as mean ± SD. Statistical significance was determined using one‑way ANOVA followed by Tukey post‑test and unpaired two-tailed Student’s t-test. **p* <0.05, ***p* <0.01, ns, not significant.


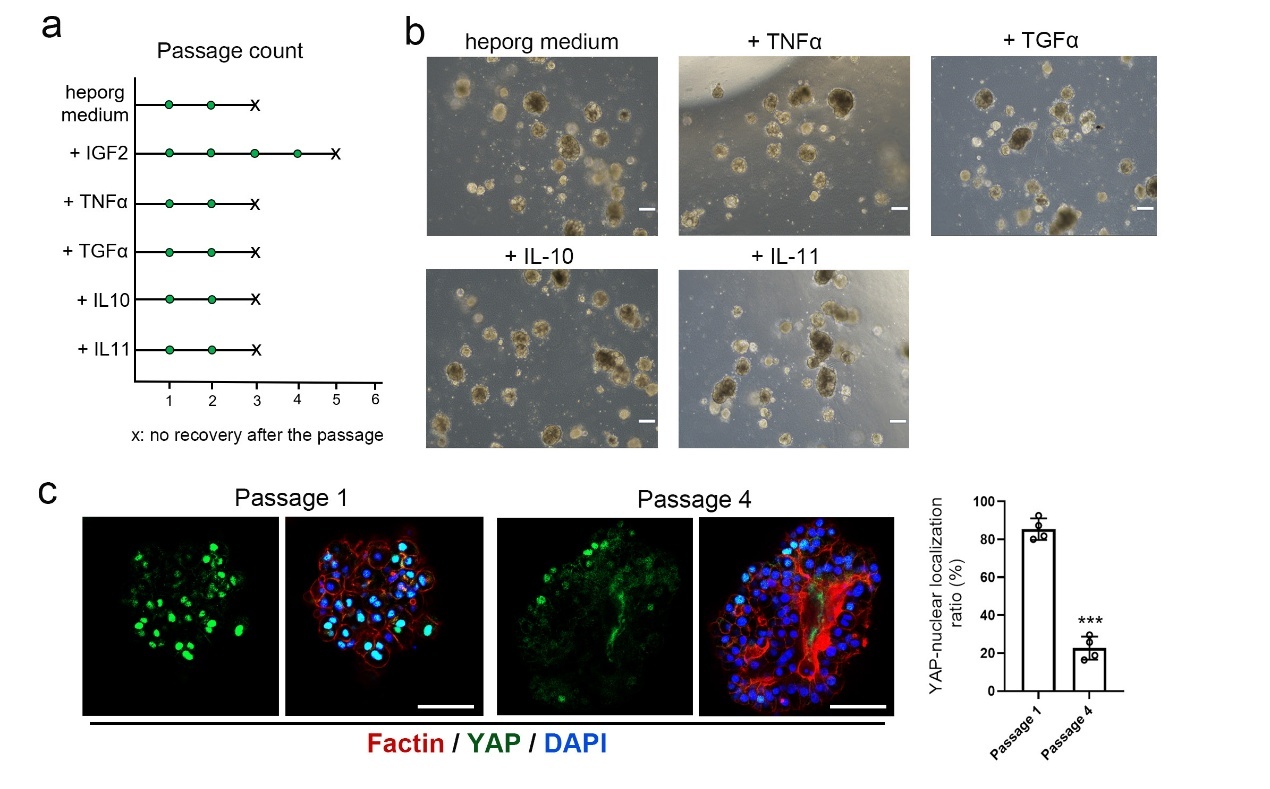


**Fig. S5 The effect of inflammatory factors and cytokines on G-heporgs**

(a) The effect of inflammatory factors and cytokines on the passage of G-heporgs. The circle indicated successful passage with subsequent organoid growth, and the x indicated passage failure. (b) Representative images of organoids treated with indicated inflammatory factors and cytokines in heporg medium. Scale bar = 100 μm. (c) Immunostaining of YAP and Factin in G-heporgs (passage 1 and passage 4) and the quantification of YAP nuclear localization. Nuclei were stained with DAPI. Scale bar = 50 μm. n = 4 biologically independent experiments. Results were presented as mean ± SD. Statistical significance was determined using unpaired two-tailed Student’s t-test. ****p* <0.001.


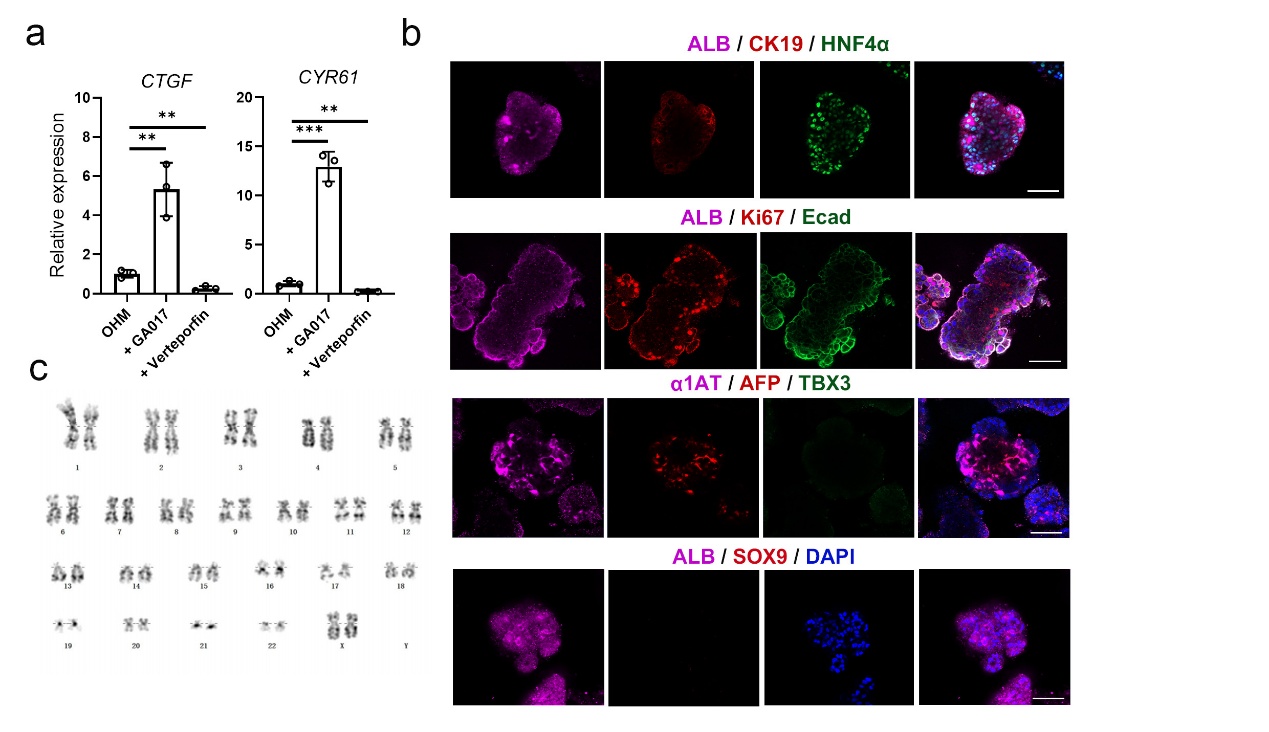


**Fig. S6 Characteristization of G-heporgs treated with GA-017**

(a) The expression of CTGF and CYR61 was analyzed by RT-qPCR in G-heporgs treated with GA017 or Verteporfin. n = 4 biologically independent experiments. (b) Immunostaining of ALB, CK19, HNF4α, α1AT, AFP, TBX3, Ki67 and SOX9 in G-heporgs treated with GA017. Nuclei were stained with DAPI. Scale bar = 50 μm. (c) Representative G-banded karyotype analysis of expandable G-heporgs at passage 10. Results were presented as mean ± SD. Statistical significance was determined using one‑way ANOVA followed by Tukey post‑test. ***p* <0.01, ****p* <0.001.


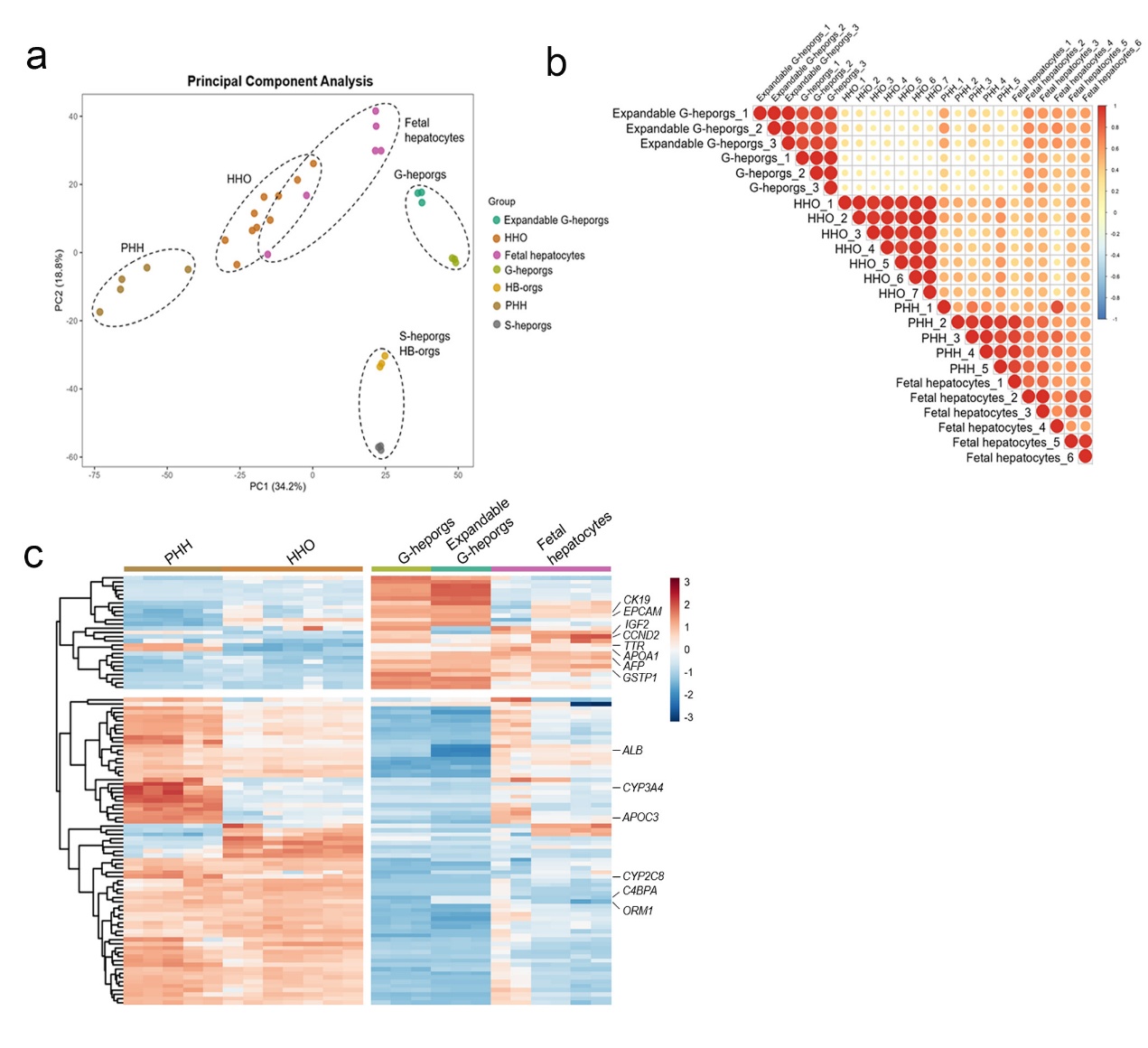


**Fig. S7 Transcriptome analysis of G-heporgs**

(a) Principal-component analysis (PCA) comparing significant genes of G-heporgs (n = 3), expandable G-heporgs (n = 3), HB-orgs (n = 3, GSE265845), S-heporgs (n = 3), PHHs (n = 5, GSE228249, GSE112866), fetal hepatocytes (n = 6, GSE112330, GSE114643, GSE137569) and human hepatocyte organoids (HHO, generated from PHHs, n = 10, GSE228249). (b) Correlation heatmap analysis. (c) Heatmap showing the top 200 significant differential expressed genes.


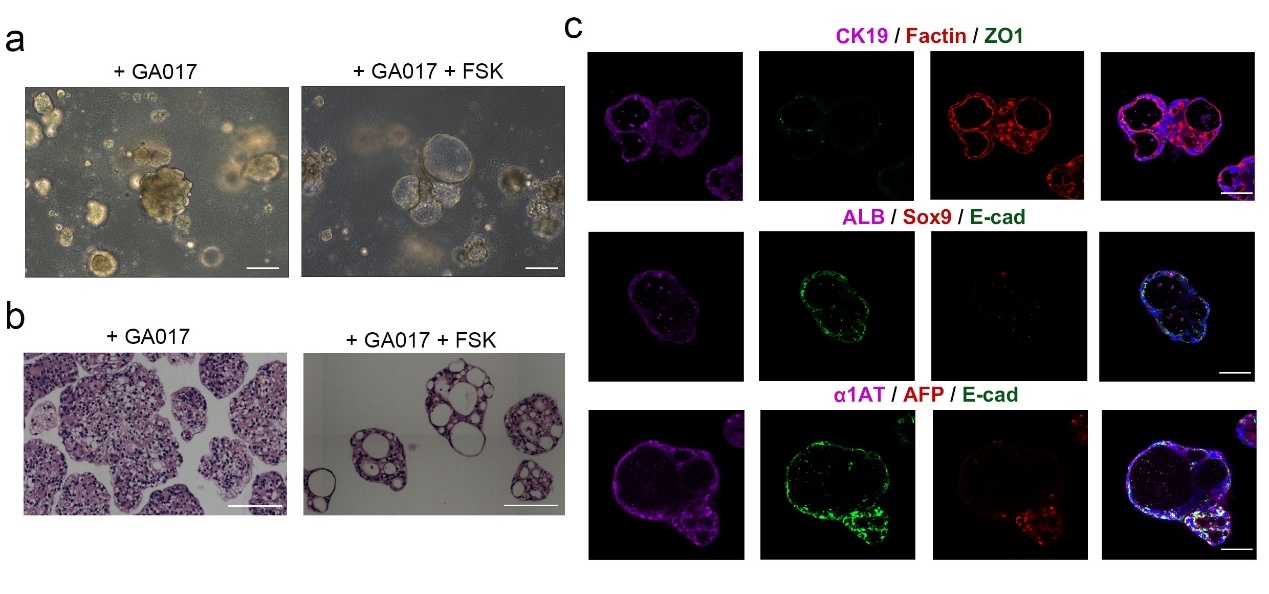


**Fig. S8 Characterization of G-heporgs**

(a) Representative images of G-heporgs treated with GA017 or FSK. Scale bar = 100 μm. (b) Representative image of H&E staining of G-heporgs treated with GA017 or FSK. Scale bar = 100 μm. (c) Immunostaining of CK19, Factin, ZO1, ALB, Sox9, E-cad, α1AT and AFP in G-heporgs treated with GA017 plus FSK. Scale bar = 50 μm.


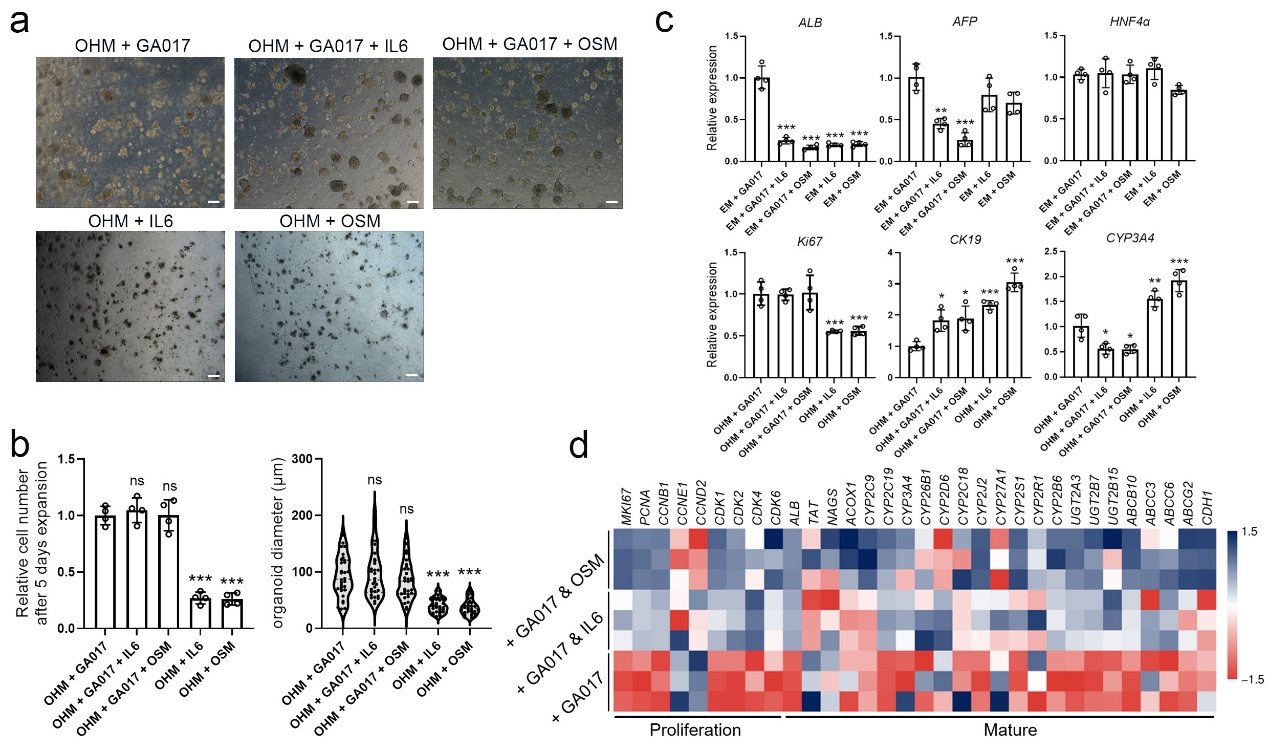


**Fig. S9 The effect of IL6 and OSM on G-heporgs**

(a) Representative images of G-heporgs treated with GA017, IL6 and OSM in optimized heporg medium (OHM). Scale bar = 100 μm. (b) Relative cell number (n = 4 biologically independent experiments) and organoid diameter (n = 30 organoids) of G-heporgs at day 5 after different treatments. (c) The expression of hepatocyte related genes was analyzed by RT-qPCR in G-heporgs after different treatments. n = 4 biologically independent experiments. (d) Heatmap of G-heporgs treated with GA017 plus IL6 or OSM in OHM for genes related to the proliferation and mature. Results were presented as mean ± SD. Results were presented as mean ± SD. Statistical significance was determined using one‑way ANOVA followed by Tukey post‑test. **p* <0.05, ***p* <0.01, ****p* <0.001, ns, not significant.

**Table S1 Primers used in this study**

| Gene | Forward | Reverse |
| --- | --- | --- |
| GAPDH | GAAGATGGTGATGGGATTTC | GAAGGTGAAGGTCGGAGTC |
| ALB | CTGCCTGCCTGTTGCCAAAGC | GGCAAGGTCCGCCCTGTCATC |
| AFP | GGGAGCGGCTGACATTAT | TGTTTCATCCACCACCAA |
| Ki67 | GAAAGAGTGGCAACCTGCCTTC | GCACCAAGTTTTACTACATCTGCC |
| CK19 | AGCTAGAGGTGAAGATCCGCGA | GCAGGACAATCCTGGAGTTCTC |
| HNF4α | GGTGTCCATACGCATCCTTGAC | AGCCGCTTGATCTTCCCTGGAT |
| α1AT | TCGCTACAGCCTTTGCAATG | TTGAGGGTACGGAGGAGTTCC |
| CYP3A4 | TCTGTGCCTGAGAACACCAGAG | TGCATGTACAGAATCCCCGGTT |
| CYP2C9 | AGGAAAAGCACAACCAACCATC | TTCAGCAGGAGAAGGAGAGCAT |
| CYP2C19 | CTGGCTGAAAGAGCTAACAGAGG | TGAACACGGTCCTCAATGCTCC |
| CYP2E1 | CCGCAAGCATTTTGACTACA | GCTCCTTCACCCTTTCAGAC |
| CYP1A1 | AGGCTTTTACATCCCCAAGG | GCAATGGTCTCACCGATACA |
| CYP1B1 | CACCAAGGCTGAGACAGTGA | GCCAGGTAAACTCCAAGCAC |
| AFP | GGGAGCGGCTGACATTAT | TGTTTCATCCACCACCAA |
| BSEP | AGCCACACAGACCAGGATGTTG | CAATGAACCGCCTCTCCTTTCC |
| MDR1 | TTGCTGCTTACATTCAGGTTTCA | AGCCTATCTCCTGTCGCATTA |
| CEBPα | CTCGAGGCTTGCCAGACCGT | GCGGGCTTGTCGGGATCTCAG |
| FXR | GGTCTCGTAGACGAAGGACTGA | TGTCTGCTCTGAGACTCAGCTC |
| IGF2 | TGGCATCGTTGAGGAGTGCTGT | ACGGGGTATCTGGGGAAGTTGT |
| IGF1R | CCTGCACAACTCCATCTTCGTG | CGGTGATGTTGTAGGTGTCTGC |
| CTGF | CTTGCGAAGCTGACCTGGAAGA | CCGTCGGTACATACTCCACAGA |
| CYR61 | GGAAAAGGCAGCTCACTGAAGC | GGAGATACCAGTTCCACAGGTC |

**Table S2 Antibodies used in this study**

| Primary antibody | | | | |
| --- | --- | --- | --- | --- |
| Antibody | Company | Product number | Ig Species | Dilution |
| Anti-GAPDH | Proteintech | 60004-1-Ig | Mouse | 30000 |
| Anti-ALB | Bethyl | A80-129A | Goat | 1000 |
| Anti-a1AT | Bethyl | A80-122A | Goat | 200 |
| Anti-YAP | CST | 14074 | Rabbit | 200 |
| Anti-Ecad | Proteintech | 20874-1-AP | Rabbit | 200 |
| Anti-ZO1 | Proteintech | 21773-1-AP | Rabbit | 200 |
| Anti-Ki67 | BOSTER | M00254-9 | Mouse | 200 |
| Anti-IGF1R | Proteintech | 20254-1-AP | Rabbit | 200 |
| Anti-ASGPR | Santacruz | sc-52623 | Mouse | 100 |
| Anti-HNF4α | CST | 3113 | Rabbit | 2000 |
| Anti-KRT19 | CST | 12434 | Rabbit | 100 |
| Anti-AFP | Thermo | MIA1305 | Mouse | 200 |
| Anti-TBX3 | Bioss | Bioss | bs-10266R | 100 |
| Anti-SOX9 | CST | 82630 | Rabbit | 500 |
| Anti-ATP7B | Santa cruz | sc-373964 | Mouse | 100 |
| Anti-MRP2 | Proteintech | 29261-1-AP | Rabbit | 500 |
| Anti-LAMP1 | CST | 9091 | Rabbit | 500 |
| Anti-Golgin-97 | Proteintech | 12640-1-AP | Rabbit | 500 |
| Anti-MDR1 | CST | 13342 | Rabbit | 200 |
| Anti-Factin (Phalloidin-iFluor 594) | Abcam | ab176757 |  | 1000 |
| Anti-AKT | CST | 4691 |  | 1000 |
| Anti-p-AKT(Thr308) | CST | 13038 |  | 1000 |
| Anti-GSK3β | CST | 12456 |  | 1000 |
| Anti-P- GSK3β(Ser9) | CST | 5558 |  | 1000 |
| Secondary antibody | | | | |
| Antibody | Company | Product number | Dilution | |
| Alexa Fluor 647-conjugated donkey anti-Goat IgG | Abcam | ab150135 | 800 |  |
| Alexa Fluor 488-conjugated goat anti-Rabbit IgG | CST | 4412 | 800 |  |
| Alexa Fluor 594-conjugated goat anti-Mouse IgG | CST | 8890 | 800 |  |
| HRP-labeled Goat Anti-Rabbit IgG(H+L) | Beyotime | A0208 | 1000 |  |
