# Supplementary figures and images for: Generation of proliferative hESC-derived grape-clustered hepatocyte organoids with multipolar architecture as regenerative counterpart via synergy of YAP and IGF2 pathways
Source: Cell Death Dis. 2026 Mar 26;17(1):381. doi: 10.1038/s41419-026-08635-y (PMC13039810; doi:10.1038/s41419-026-08635-y)

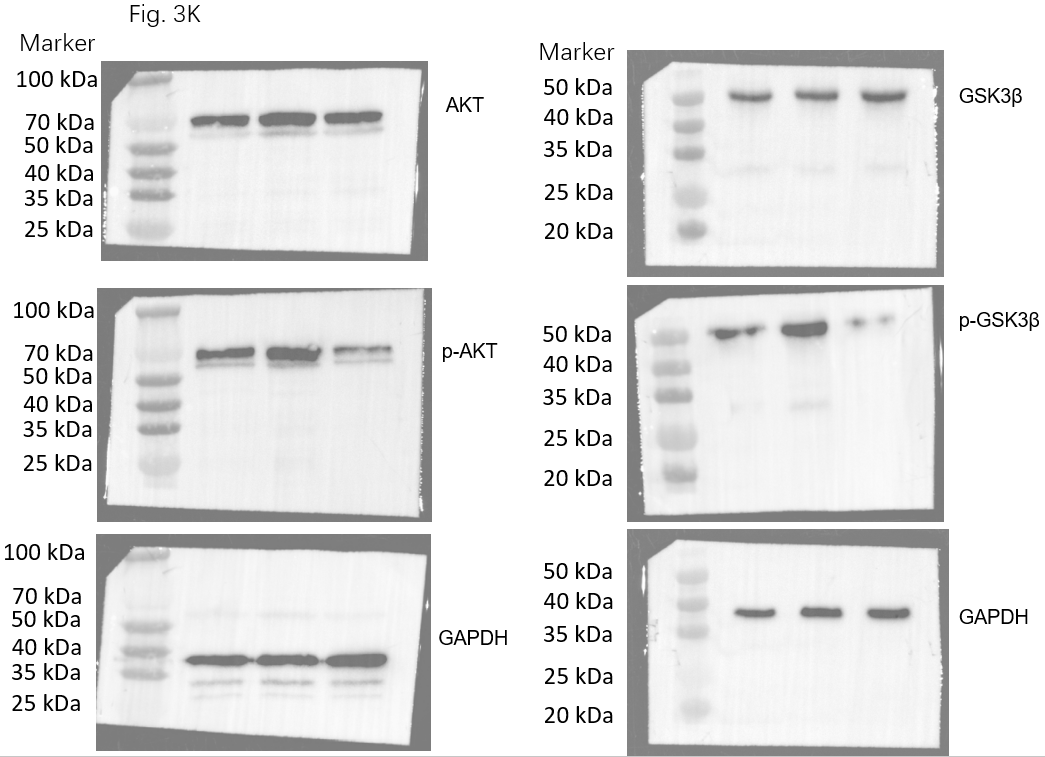

Supplement: Supplementary file 2 — Original WB images [file 41419_2026_8635_MOESM2_ESM.docx]
